# Whole Genomic Constellation of Avian Reovirus Strains Isolated from Broilers with Arthritis in North Carolina, USA

**DOI:** 10.3390/v15112191

**Published:** 2023-10-31

**Authors:** Islam Nour, Sonsiray Alvarez-Narvaez, Telvin L. Harrell, Steven J. Conrad, Sujit K. Mohanty

**Affiliations:** United States Department of Agriculture, Agricultural Research Service (USDA-ARS), US National Poultry Research Center, Athens, GA 30605, USA; islam.mohamed@usda.gov (I.N.); sonsiray.alvareznarvaez@usda.gov (S.A.-N.); telvin.harrell@usda.gov (T.L.H.); steven.conrad@usda.gov (S.J.C.)

**Keywords:** ARV, genome, phylogenetic analysis, amino acid, recombination

## Abstract

Avian reovirus (ARV) is an emerging pathogen which causes significant economic challenges to the chicken and turkey industry in the USA and globally, yet the molecular characterization of most ARV strains is restricted to a single particular gene, the sigma C gene. The genome of arthrogenic reovirus field isolates (R18-37308 and R18-38167), isolated from broiler chickens in North Carolina (NC), USA in 2018, was sequenced using long-read next-generation sequencing (NGS). The isolates were genotyped based on the amino acid sequence of sigma C (σC) followed by phylogenetic and amino acid analyses of the other 11 genomically encoded proteins for whole genomic constellation and genetic variation detection. The genomic length of the NC field strains was 23,494 bp, with 10 dsRNA segments ranging from 3959 bp (L1) to 1192 bp (S4), and the 5′ and 3′ untranslated regions (UTRs) of all the segments were found to be conserved. R18-37308 and R18-38167 were found to belong to genotype (G) VI based on the σC analysis and showed nucleotide and amino acid sequence identity ranging from 84.91–98.47% and 83.43–98.46%, respectively, with G VI strains. Phylogenetic analyses of individual genes of the NC strains did not define a single common ancestor among the available completely sequenced ARV strains. Nevertheless, most sequences supported the Chinese strain LY383 as a probable ancestor of these isolates. Moreover, amino acid analysis revealed multiple amino acid substitution events along the entirety of the genes, some of which were unique to each strain, which suggests significant divergence owing to the accumulation of point mutations. All genes from R18-37308 and R18-38167 were found to be clustered within genotypic clusters that included only ARVs of chicken origin, which negates the possibility of genetic pooling or host variation. Collectively, this study revealed sequence divergence between the NC field strains and reference ARV strains, including the currently used vaccine strains could help updating the vaccination regime through the inclusion of these highly divergent circulating indigenous field isolates.

## 1. Introduction

Avian reoviruses (ARVs) are pathogens of domestic poultry as well as of wild birds. Infection leads to a wide spectrum of clinical presentations, including tenosynovitis/arthritis immunosuppression, enteric disease, malabsorption and runting–stunting syndrome [1,2]. Domestic avian species are mostly vulnerable to ARV infection. Meat-type chickens are, however, more susceptible to viral arthritis/tenosynovitis caused by ARV than are other species [3] such as broiler/layer breeders [3,4], duck [5,6,7], geese [8,9], turkeys [10,11], quails [12,13,14] and pigeons [15]. 

The pathological outcomes and the subclinical disease presentation of ARV cause reduced productive parameters, as indicated by a lack of flock uniformity, lower weight gain, diminished feed conversion rates, more lameness-related condemnations in the processing plants and welfare concerns [16], resulting in tremendous economic losses to the poultry industry [17]. The 2015 reovirus tenosynovitis outbreak in California impacted 14–47-day-old broilers and resulted in high morbidity and mortality. These high rates could be due to ARV isolates that escaped vaccine efforts despite constant immunization regimes since the 1970s using classical vaccine strains (S1133, 1733 and 2408). Indeed, these vaccine strains have shown inconsistencies in infection control owing to positive selection pressure due to the generated antibodies against these vaccine strains as well as the high mutation rate and recombination events characteristic of RNA viruses [18]. The emergence of immune-escaping strains that circumvent vaccine immunity necessitates the prompt detection and typing of newly circulating strains that could help in autogenous vaccine generation [19,20,21]. Therefore, characterizing the circulating ARV strains causing disease in the field is the initial step to reach the proper control strategies. Countries such as the USA [21,22,23,24], Canada [25,26] and China [17] have already documented their attempts at detecting and typing the circulating ARV strains in some of their territories. 

ARV belongs to the *Orthoreovirus* genus, subfamily *Spinareovirinae* of the *Reoviridae* family [27,28]. ARV is a non-enveloped virus with a 70–80 nm icosahedral double-layered capsid containing a segmented double-stranded RNA (dsRNA) genome [29]. The 10-segment genome is divided into three classes, based on their electrophoretic mobility: L-class (large segments; L1–L3), M-class (medium segments; M1–M3) and S-class (small segments; S1–S4) [30,31]. S2, S3, S1, M1, M2, L1, L2 and L3 encode eight structural proteins: σA, σB, σC, μA, μB, λA, λB and λC, respectively. Four nonstructural proteins encoded by S1 (P10 and P17), S4 (σNS) and M3 (μNS) are not detected in the mature virions [32]. Interestingly, the 5′ untranslated regions (UTRs) and 3′ UTRs of the ARV genomic segments are highly conserved sequences: 5′-GCUUUUU-3′ and 5′-UCAUC-3′, respectively [33]. Currently, intensive attention is paid to the S1 segment since it encodes the viral attachment and major immunogenic protein σC that plays a crucial role during the early infection stages and provokes type-specific neutralizing antibodies [28,34]. Consequently, σC sequencing-based analysis has been frequently used as a genetic index for ARV characterization and classification [21,24,35,36]. To date, five, six and seven genotypes have been reported according to Lu, Kant and Sellers’s classifications, respectively [24,28,37]. Despite the frequent emergence of novel ARV strains and the associated economic burden, molecular characterization is typically performed only for the σC gene. Herein, we report the complete genome sequence of two field strains, R18-37308 and R18-38167, isolated from 58- and 60-day-old broilers in North Carolina (NC), USA, by using long-read next-generation sequencing (NGS). Moreover, recombination analysis was conducted to explore any possible emergence from previous parental strains.

## 2. Materials and Methods

### 2.1. ARV Strain Isolation and Propagation

The ARV field strains R18-37308 and R18-38167 used in this study were isolated from the tendons and synovial tissues of sick broilers showing arthritis/tenosynovitis from two NC commercial broiler flocks at 58 and 60 days of age in 2018. Virus isolation and propagation were carried out in LMH cells (ATCC CRL-2113) [38]. Following the virus-mediated syncytia formation and complete destruction of the cell monolayer, three cycles of freeze-thawing were carried out. The complete cell lysate was centrifuged at 5000 rpm for 30 min at 4 °C to remove cell debris, followed by ultracentrifugation at 36,000× *g* for 2 h at 4 °C. The supernatant was discarded, and the obtained pellet was suspended in 1 mL of PBS and stored at −80 °C until further use.

### 2.2. Genomic RNA Extraction and Reverse Transcription 

RNA was extracted from 140 μL of each virus pellet using the Qiagen viral mini-RNA isolation kit (Qiagen, Germantown, MD, USA) following the manufacturer’s protocol. Total RNA (about 1 µg) was reverse transcribed into cDNA in a 20 μL reaction volume containing 200 ng of random hexamers, 1 µL of 10 mM dNTP mix, 5 μL of 5× buffer, 1 μL of 0.1 M DTT and 1 μL of Superscript III reverse transcriptase (Invitrogen, Waltham, MA, USA). The reaction mixture was incubated at 25 °C for 5 min followed by 50 °C for 60 min and heat inactivation at 70 °C for 15 min. The reaction mixture was then treated with RnaseH (1 μL) for 20 min at 37 °C.

### 2.3. ARV Full-Length Genome Amplification

For each virus, the whole genome of each segment was amplified by PCR using ARV-specific tagged primers designed for this study (primer sequences will be provided upon request). Briefly, a 25 μL PCR mixture was obtained using 2 μL of cDNA template, 500 nM each of the forward and reverse primer, 300 μM dNTPs, 5 μL of 5× LongAmp Taq Reaction Buffer and 1 μL (2.5 U) of LongAmp Taq DNA Polymerase (NEB, Ipswich, MA, USA). The PCR was performed at 94 °C for 3 min, followed by 35 cycles of 94 °C for 30 s and 65 °C for 4 min 30 s, with a final extension at 65 °C for 10 min in a Veriti^TM^ Fast Thermal Cycler (Applied Biosystems^TM^, San Francisco, CA, USA, Cat. 4375305). A 1% agarose gel was used to visualize the yielded amplicons. Amplicons of the corresponding size were gel-purified using the PureLink Quick Gel Extraction Kit (Invitrogen, USA) according to the manufacturer’s instructions. 

### 2.4. ARV Whole Genome Sequencing and Raw-Data Processing

Accurate quantification of each purified segment was conducted using the QubitTM dsDNA HS assay kit (Invitrogen, USA). Whole genome sequencing was conducted using Oxford Nanopore Technologies’ (ONT’s) long-read sequencing (Eurofins Genomics, Louisville, KY, USA) from a 300 ng sample of purified PCR product. Library preparation was conducted for each individual segment separately (i.e., twenty library preparations for the two NC isolates) using the Rapid Barcoding Kit 24 V14 (SQK-RBK114.24). Priming was carried out using Flow Cell Priming Kit V14 (EXP-FLP004) before loading the SpotON R10.4.1 flow cells (FLO-MIN114). Visualization and processing of the long-read sequencing data was carried out using NanoPack [39]. NanoFilt was used for NGS-generated read filtering and trimming, depending on the length, mean quality and GC content of each read. The sequencing reads obtained were quality checked using NanoStat and NanoPlot. NanoStat generated a complete statistical data analysis, including the mean and median read quality, mean and median read length, read length N50, number, percentage and megabases of reads above quality cutoffs, whereas NanoPlot generated the quality control graphs involving read-length histograms, bivariate plots and cumulative yield plots. The reads that passed the quality check were de novo assembled using Geneious Prime (Version 2023.0.1), and a consensus sequence was generated.

### 2.5. Phylogenetic Analyses 

ARV amino acid (aa) sequences of the 12 proteins of each virus strain were subjected to various phylogenetic analyses using the MEGA 11 software [40]. For the R18-37308 and R18-38167 genotyping, the σC portion of the S1 segment of both isolates were extracted manually using Geneious Prime 2022.1.1 (https://www.geneious.com, last accessed on 15 October 2023) together with the σC sequences of 68 isolates representing well-defined genotypes according to Kant’s classification [28]. The phylogenetic relatedness of the 70 sequences was estimated using the best-fitting substitution model and the Pycno-1 isolate (NCBI accession number: AB914766) as an outgroup. First, sequence alignments were generated using ClustalW, with the default settings of an opening penalty of 15 and an extension penalty of 6.66. A phylogenetic tree was constructed using the maximum likelihood method and the best-fit amino acid substitution model following the minimum Bayesian information criterion. A total of 56 amino acid substitution models were tested, and the Akaike information criterion, maximum likelihood value (lnL), corrected value and the number of parameters used (including branch lengths) were measured for each of the models using MEGA 11. The reliability of the phylogenetic tree was estimated by the bootstrapping of 1000 replicates. The names and accession numbers of all the ARVs used in this study are summarized in Table S1. Prediction of the open reading frames (ORFs) was carried out using the NCBI’s website (https://www.ncbi.nlm.nih.gov/orffinder/, last accessed on 1 September 2023). Following the ORF prediction, phylogenetic analysis was conducted for each amino acid sequence of the remaining 11 proteins against 16–18 reference sequences, following the same methodology described for the σC phylogeny. Reference sequences were selected to cover all the genotypes retrieved from the σC analysis and should have all the genomic segments available for a unified characterization of genotypic clusters (i.e., using the same isolates for the entire coding-region-based phylogenetic analyses). Moreover, any reference sequence that masked significant variations between different isolates were omitted for an enhanced and unbiased phylogenetic analysis. For a whole genome comparison of our field strains with other reference strains, full genomic sequences of one turkey strain (22342), one partridge strain (D1007), one bulbul strain (Pycno-1) and seven ARV reference strains (S1133, 1733, 01224A, PHC-2020-0545, 3211-V-02, AVS-B and LY383) were retrieved from GenBank and used for comparative analysis using the mVISTA online platform (http://genome.lbl.gov/vista/mvista/submit.shtml, last accessed on 27 September 2023) [41].

### 2.6. Detection of Recombination Incidence

Potential recombination events in the ARV genomic segments were assessed using RDP4 version 4.101 [42]. RDP4 analysis was carried out based on the complete genomic sequence of each ARV gene, using seven algorithms, including RDP, BootScan, GENECONV, Chimera, SISCAN, maximum chi square and 3SEQ. A putative recombination event was passed to subsequent analysis only if it was plausibly defined by at least four of the above-mentioned seven algorithms.

## 3. Results

### 3.1. R18-37308 and R18-38167 Sequencing Data

The 20 segments that constitute the ARV isolates R18-37308 and R18-38167 were sequenced using ONT long-read sequencing. A total of 13,941 reads for R18-37308 and 13,169 for R18-38167 passed the quality filter (Phred scores > 12) and were included in the genome assembly process. These reads translated to 17,160,962 bp and 15,638,274 bp, respectively, and an average sequencing depth of X749 and X729 (Table S1).

### 3.2. σC-Based Genotyping Determined That R18-37308 and R18-38167 Clustered with Genotype VI

A phylogenetic analysis that included the σC sequences of the novel isolates R18-37308 and R18-38167 together with the 68 σC sequences of well-defined representatives of the current genogroups [28] was performed to determine the R18-37308 and R18-38167 σC genogroup. 

**Figure 1:**
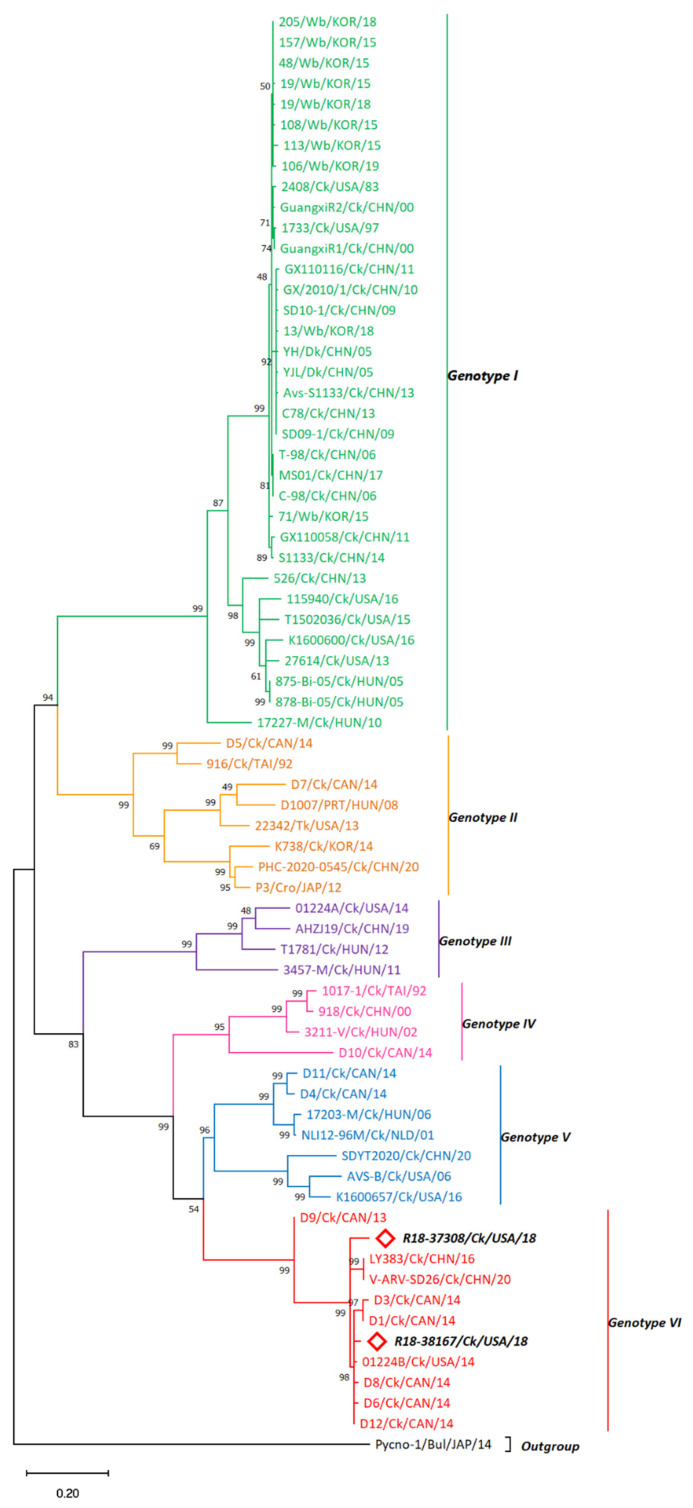
Phylogenetic analysis of σC amino acid (aa) sequences using the Maximum Likelihood method and the Jones et al. w/freq. model [43]. The tree with the highest log likelihood (−9064.39) is shown. Evolutionary rate differences were modeled using a discrete gamma distribution among sites (five categories (+G, parameter = 1.8736)). The rate variation model allowed for some sites to be evolutionarily invariable ([+I], 9.17% sites). A total of 327 positions exist in the final dataset. The accession numbers of sequences used for phylogenetic analysis are shown in Table S3.

The σC-based phylogenetic analysis grouped the sequences within six distinct clusters (G I-G VI) sharing > 70% aa identity. The σC of R18-37308 and R18-38167 strains depicted 84.91 (83.43)–94.8 (94.47) and 87.46 (86.8) %–98.47 (98.46) % nucleotide (amino acid) sequence identity, respectively, toward G VI strains. In terms of host range, the vaccine strains showed a tendency to cluster within G I, together with wild birds and duck reovirus strains (Figure 1). Turkey (Tk) and Tk-like reovirus strains were grouped within G II, whereas reoviruses of chicken origin were prevalent within all genotypes. The NC broiler reovirus strains R18-37308 and R18-38167 were clustered with chicken reovirus strains within G VI, showing no host variation (Figure 1). R18-37308 and R18-38167 were found to be more related to the Canadian strain D12 isolated in 2014 (*d* = 0.0563 and 0.01509, respectively; Table S2) than to each other (*d* = 0.0713067). R18-37308 and R18-38167 showed less aa sequence homology (93.55%) to each other compared with their homology with the D12 sequence.

### 3.3. Whole Genome Organization of the R18-37308 and R18-38167 Strains Show Conserved Terminal UTRs and the Lack of a Common Ancestor

The sequencing results disclosed the full-length genome of the ARV strains R18-37308 and R18-38167, consisting of 23,494 bp. The terminal 5′ and 3′ UTRs were conserved among all the genomic segments and were similar to those in the reference strains, involving GCUUUU(U) and UCAUC, respectively. The length of the 5′ UTR ranged from 12 nt (L3 and M1 segments) to 30 nt (S1 and S2 segments), whereas the 3′ UTR ranged from 33 nt (S1 segment) to 98 nt (M2 segment). The whole genome annotation of the isolates is displayed in Table 1. Following genomic annotation of the NC strains, phylogenetic analyses of all the genomic segments was conducted using unified reference sequences (Table S4), selected according to the criteria mentioned above (Section 2.5). 

#### 3.3.1. The L-Class Genomic Segments 

**Figure 2:**
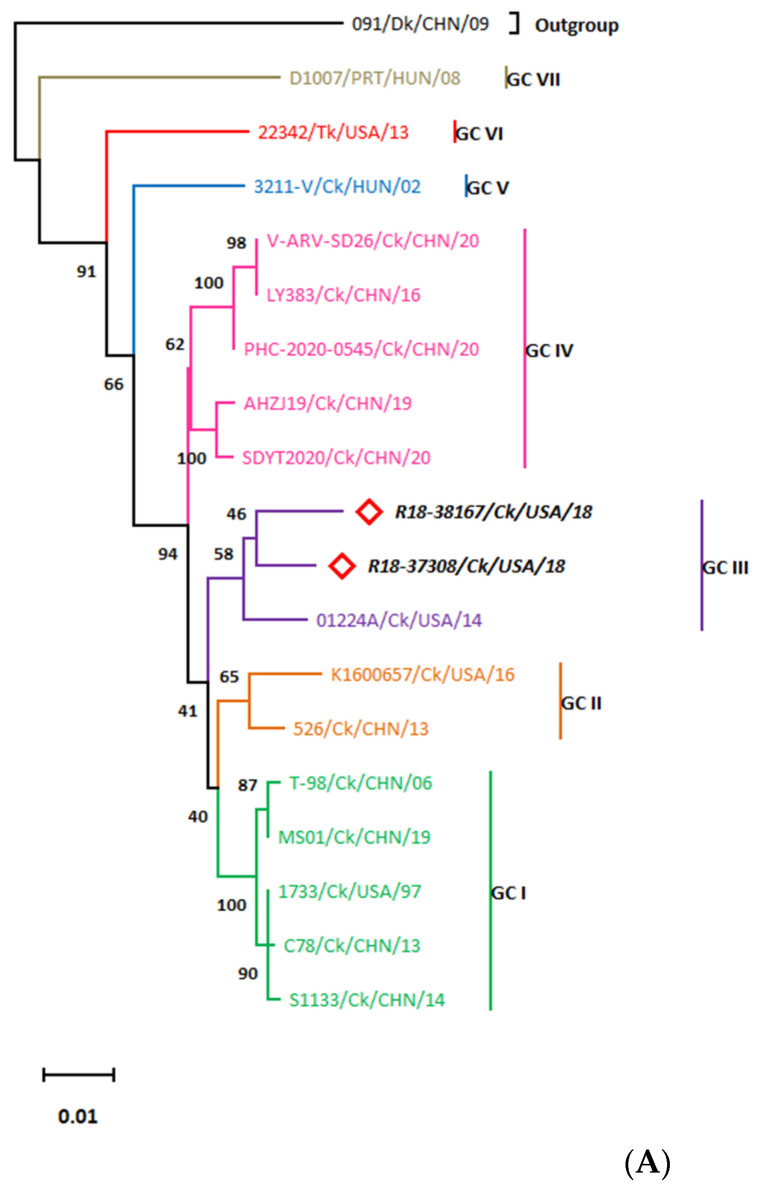

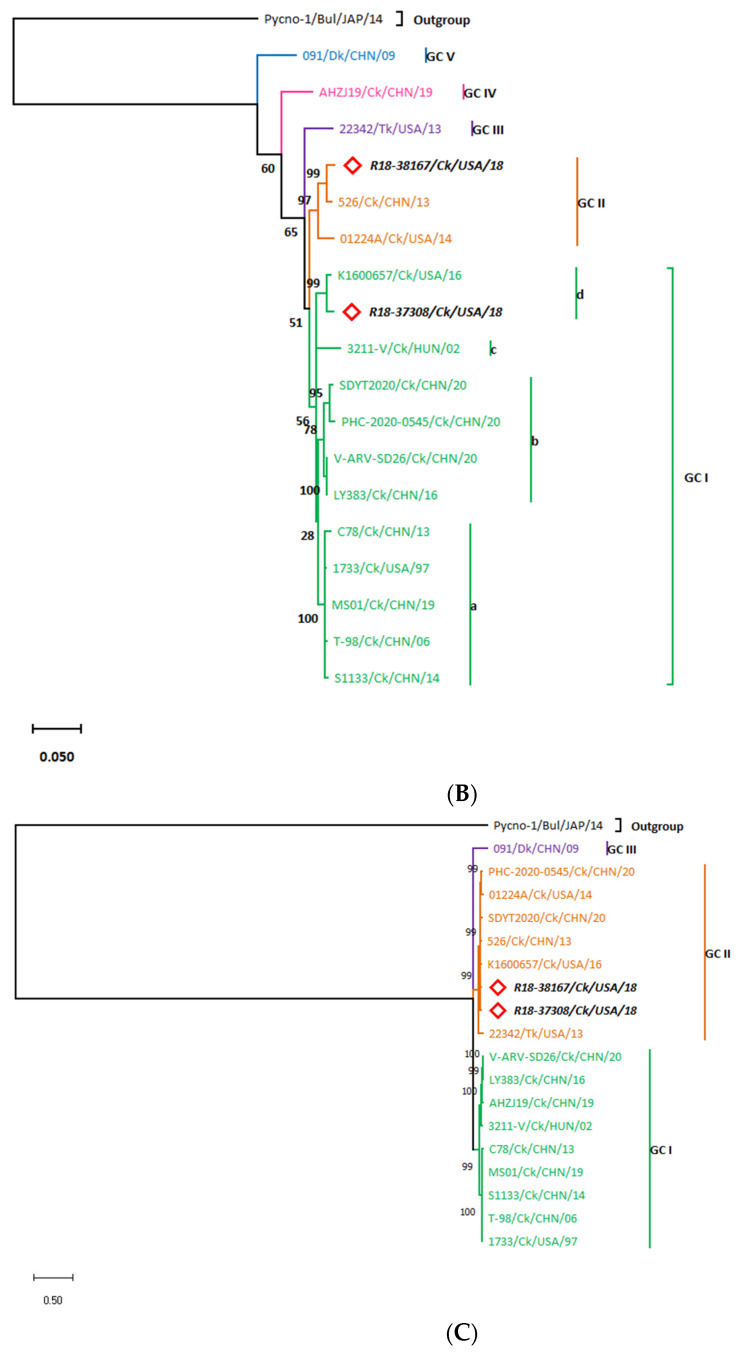

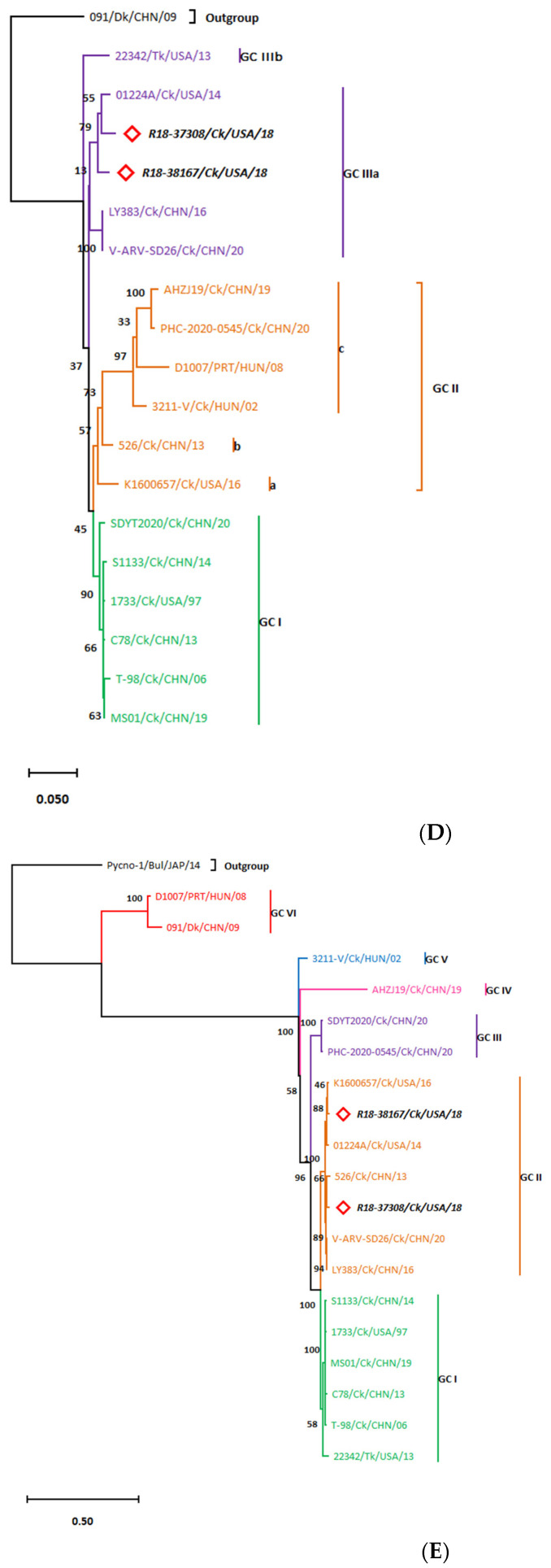

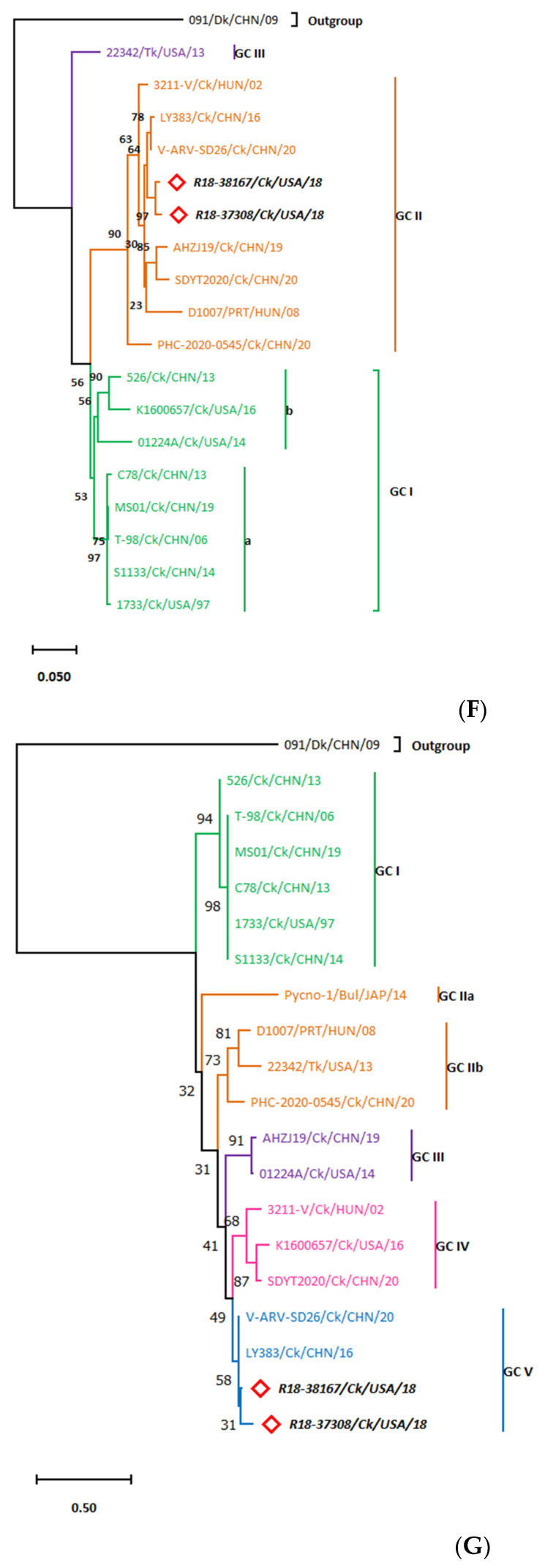

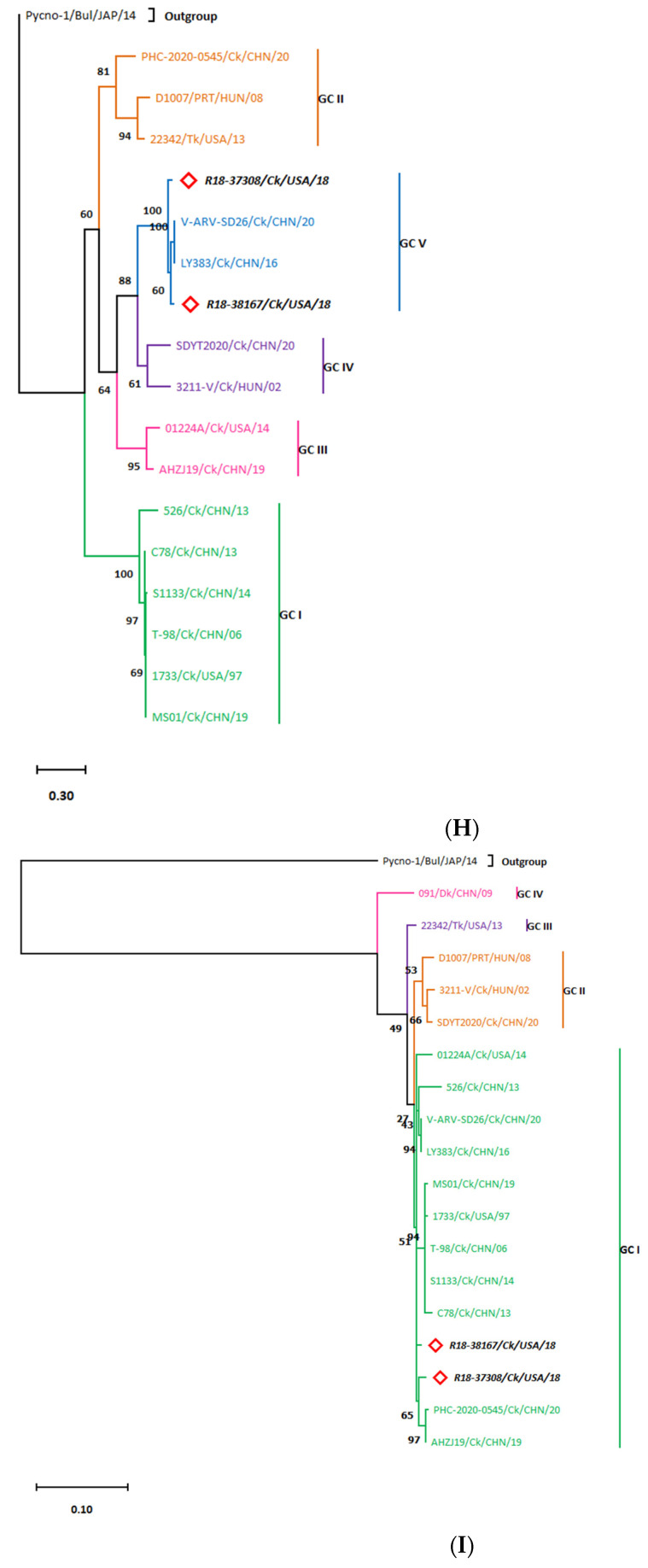

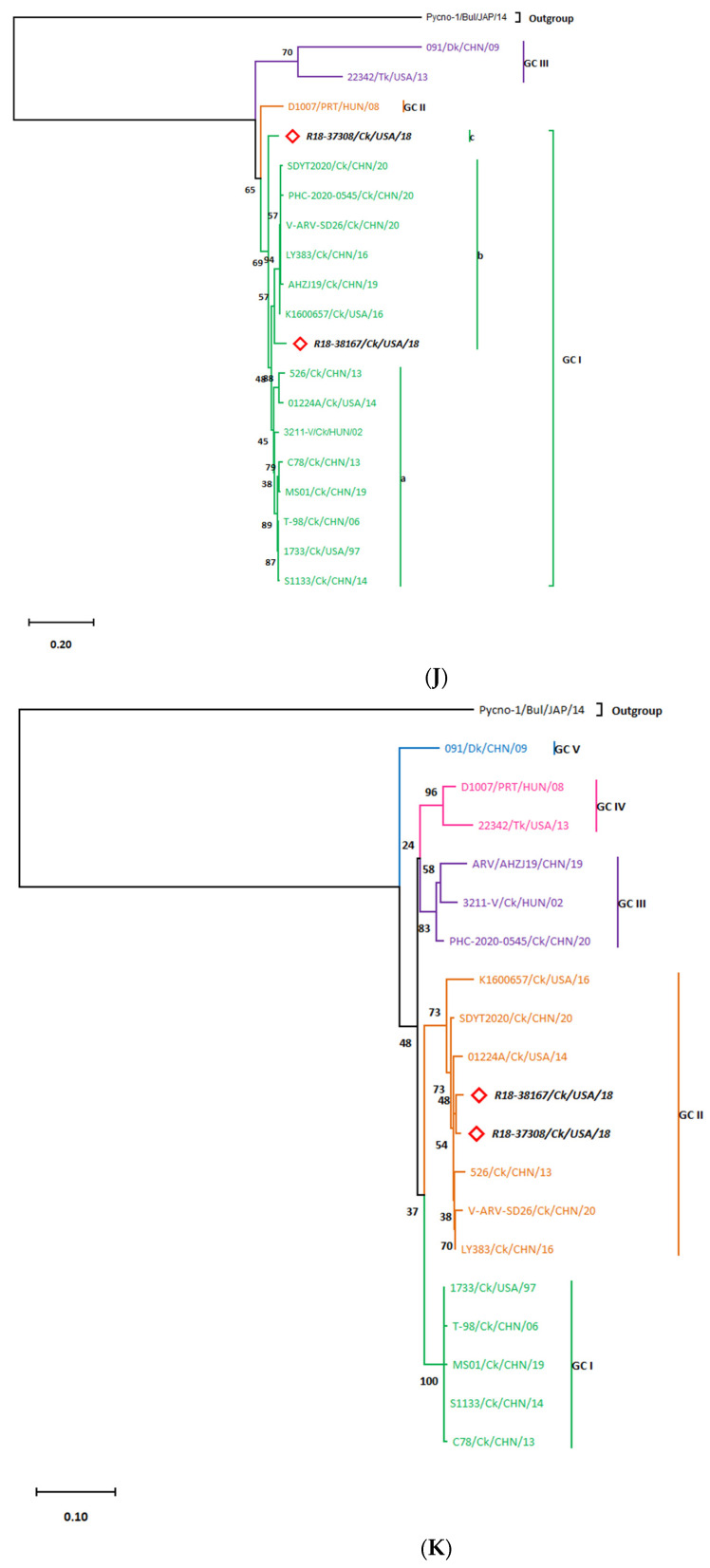
Phylogenetic analysis of the NC strains’ aa sequences using the Maximum Likelihood method and the method of Jones et al. (**A**) Lambda A ^1^, (**B**) Lambda B ^2^, (**C**) Lambda C ^3^, (**D**) µA ^2^, (**E**) µB ^2^, (**F**) µNS ^4^, (**G**) p10 ^5^, (**H**) p17 ^2^, (**I**) σA ^2^, (**J**) σB ^2^ and (**K**) σNS ^2^. Notes: ^1^ JTT + G + F model; ^2^ JTT + F model; ^3^ mtREV24 + G + F model; ^4^ JTT + G + I model; ^5^ LG + G model. Accession numbers of the sequences used for the phylogenetic analysis are shown in Table S19.

The Lambda A (λA) aa sequence of the R18-37308 and R18-38167 strains encoded by the L1 segment was compared with that of 17 strains (Table S3). The phylogenetic analysis of λA demonstrated seven genotypic clusters with >97.9% aa identity within each cluster. Although R18-37308 shared 98.14% aa sequence homology with R18-38167, R18-37308 was more closely related to 01224A (*d* = 0.019016; Table S5) than to R18-38167 (*d* = 0.02042). These strains constituted a single branch in the phylogenetic tree, genotypic cluster (GC) III, that is distinguishable from turkey, partridge and duck ARVs (Figure 2A). Amino acid analysis of R18-37308 and R18-38167 showed 26 and 30 amino acid substitution events, respectively. The A228T aa substitution was characteristic of the two new isolates, whilst D38E defined GC III strains (Table S6). Moreover, R18-37308 contained four unique aa substitutions (I31D, V45A, T57A and V112M), while R18-38167 contained five (A78K, K116R, S138A, T300N and K313N). 

On the other hand, the R18-37308 and R18-38167 L2 genomes showed less aa sequence homology with each other (95.95%) when compared with that for the L1 genome. Moreover, the phylogenetic analysis of Lambda B, encoded by the L2 segment, depicted five genotypic clusters: (GC I–V; aa identity > 96%) with 4 sub-lineages (a–d) within the GC I cluster (Figure 2B). R18-37308 was found to be closely related to K1600657 (isolated from the USA in 2016; *d* = 0.01081; Table S7) within GC Id, while R18-38167 showed a close relationship to the Chinese strain 526 (*d* = 0.01221) and clustered within GC II. Both GC I and GC II contained only ARVs of chicken origin. The R18-37308 and R18-38167 aa analysis revealed 34 and 44 aa substitutions, respectively. R18-37308 contained six unique aa substitutions, including K26T, Q84R, D89E, V293I, H551Y and V929S (Table S6). R18-38167 showed two substitution events characteristic of GC II, which were M638V and E1061D; however, R18-37308 comprised P88D, S152T, T233A and N928T aa substitutions defining the GC Id cluster. 

Lambda C aa sequences displayed three distinct clusters (GC I-III; Figure 2C) with >91% aa homology within each cluster. The NC strains were more closely related to each other (97.43% identity, *d* = 0.027431; Table S8) than to the reference ARVs and belonged to GC II. Although GC II included the turkey strain 22342, the NC isolates were found to be evolutionarily distant from the 22342 strain (*d* = 0.099). R18-37308 showed two unique aa substitutions (P269S and T1053A), whilst R18-38167 demonstrated five distinct mutations, including T23S, N293S, S472A, D700N and I958V (Table S6). Moreover, the triplet DRN at aa 48–50 differentiated the GII strains of chicken origin from those of turkey origin (GQD).

#### 3.3.2. The M-Class Genomic Segments

The µA sequences, encoded by the M1 segment, indicated four genotypic clusters with greater than 92.7% aa identity (Figure 2D), of which GC II had three subclusters (a–c). R18-37308 and R18-38167 aa sequences were more closely related to ARV 01224A (*d* = 0.021967 and 0.0235, respectively; Table S9) than toward each other (*d* = 0.0288) and grouped within GC IIIa, which was distinguishable from the partridge isolate and the duck strains. Moreover, R18-37308 showed high aa sequence identity to the R18-38167 strain (97.68%). Interestingly, the 01224A strain shared a specific mutation, Y377H, with our strains. On the other hand, R18-37308 displayed four distinct aa substitutions involving T55I, A393T, K417R and S598T (Table S10), whereas R18-38167 contained other four unique aa substitutions (T44P, A170I, P280L and S598G).

The µB aa sequences, encoded by the M2 segment, showed six distinct branches referred to as GC I-VI (Figure 2E), with more than 93% aa homology within each genotypic cluster. R18-37308 and R18-38167 showed a clear clustering within GC II that includes only chicken ARVs, unlike GC I (containing turkey ARV) and GC VI (containing duck and partridge strains). R18-37308 exhibited an aa sequence homology of 96.75% with the R18-38167 strain. R18-37308 and R18-38167 were found to be evolutionarily related to the V-ARV-SD26 (*d* = 0.0162866, Table S11) and K1600657 (*d* = 0.0104486) strains, respectively. The µB aa analysis demonstrated five aa substitutions characteristic of GC II, namely, G80S, N139S, R160K, E234D and A272T (Table S10). R18-37308 displayed three unique aa substitutions (S460L, V484T and I593V) and R18-38167 only one (S109T).

The µNS sequences, encoded by the M3 segment, disclosed three phylogenetic branches (GC I-III; aa identity > 93.5% within each GC), and GC I was composed of two subclusters: a (containing vaccine strains) and b; Figure 2F). The present study strains depicted the closest relationship to each other (*d* = 0.011178; Table S12) and were clustered within GC II. Although G II contained the partridge ARV (D1007) strain, it was evolutionarily distant from the R18-37308 and R18-38167 strains (*d* = 0.0629478 and 0.061279). Furthermore, among the M-class segments, the µNS of R18-37308 strain showed the highest aa identity to R18-38167 (98.89%) compared with that of µA and µB. Interestingly, the aa analysis of µNS sequences revealed that the NC strains shared two distinctive mutations, V590M and S618L (Table S10), and that the A300S and S253T aa substitutions were unique to R18-37308 and R18-38167, respectively.

#### 3.3.3. The S-Class Genomic Segments

The whole genome sequencing results revealed the tricistronic nature of the S1 segment, with partially overlapping ORFs encoding P10, P17 and σC similar to those in chicken ARV strains (P10, nt 31–321; P17, nt 293–733; and σC, nt 630–1610). However, a mutation in the *p10* gene start codon of the NC ARVs led to the initiation of translation at the next start codon and, consequently, the loss of the initial three amino acids at the N-terminus. Phylogenetic analysis of the P10 sequences revealed five genotypic clusters (GC I–V; Figure 2G), with aa homology of >79% within each GC. R18-37308 and R18-38167 showed obvious clustering within GC V, which includes only chicken ARVs. Although the R18-37308 strain displayed 93.81% aa sequence homology to the R18-38167 strain, R18-38167 strain was found to be more closely related to two Chinese isolates, LY383 and V-ARV-SD26 (*d* = 0.01389; Table S13), than to R18-37308 (*d* = 0.06560). The M93A aa substitution that was found to be characteristic of GC V (Table S14) was present in the newly characterized strains. Notably, only R18-37308 contained two additional unique mutations in P10, namely, A67V and V77A. The P17 sequences clustered into five distinct branches (GC I-V, with aa identity of >76%; Figure 2H), with R18-37308 and R18-38167 grouping within the GC V cluster. Similar to the findings in P10, the R18-38167 strain demonstrated a closer relationship to the LY383 and V-ARV-SD26 strains (*d* = 0.03802; Table S15) than to R18-37308 (*d* = 0.05696). Also, the P17 aa sequence of the R18-37308 strain shared 93.87% homology with the R18-38167 P17 sequence. Moreover, aa analysis revealed three distinctive aa substitutions to be found in the P17 sequence of R18-37308 (G34N, I96V and L99D). In terms of the σC aa analysis, R18-37308 and R18-38167 showed six and seven unique aa substitutions, respectively. The R18-37308 strain contained aa mutations at positions I54T, T101V, T119E, T120K, S133A and D144G (Table S14), whilst the R18-38167 displayed aa substitutions involving I33V, S108H, T119K, V121A, G123V, K150E and T256V. The σC protein contained a highly conserved motif at aa residues 319-327at the carboxyl end (NH_2_-LTVRTGIDT-COOH) that was detected in all ARVs, with a single aa substitution L319I in the K1600657 and SDYT2020 isolates.

The σA aa sequences, encoded by the S2 segment, showed four genotypic clusters (GC I–IV; Figure 2I) with an aa identity of >97.5% within each GC. Our study strains displayed a closer relationship to the LY383 and V-ARV-SD26 strains (*d* = 0.00947; Table S16) than to R18-37308 (*d* = 0.01426) and clustered within σA-GC I, which contained chicken ARVs exclusively. Still, the σA aa sequence of R18-37308 was highly homologous (98.56%) to the R18-38167 strain, but each of them had their own unique substitutions (R18-37308 carried S119P, T229N and L365I, while R18-38167 presented with V225I and P315S; Table S14).

The σB sequences, encoded by the S3 segment, demonstrated three phylogenetic branches (GC I-III, Figure 2J), with GC I including three subclusters (a–c). R18-38167 was clustered within GC Ic and was closely related to K1600657 (GC Ib—isolated from the USA in 2016; *d* = 0.054046; Table S17), whereas R18-37308, clustered within GC Ib, was closely related to the T-98 strain (within the vaccine strains cluster; *d* = 0.06027). This could also be noticed in the low sequence identity of the σB aa sequence of the R18-37308 strain with that of R18-38167 strain (93.18%). Moreover, the σB aa analysis revealed 4 and 7 aa substitutions that were unique to R18-37308 (T34A, S144G, G348D and V350T) and R18-38167(K21Q, N127K, D134G, S193A, L194V, S198A and A344P), respectively (Table S14). Interestingly, S229A and H277Q were found to occur only in GC Ib.

The σNS sequences, encoded by the S4 segment, depicted five distinct clusters (GC I–V, Figure 2K), with aa identity of more than 94.5% within each GC. R18-37308 and R18-38167 were grouped within GC II, which contained only chicken ARVs. R18-37308 was more closely related to the LY383 strain (*d* = 0.011158; Table S18) than to R18-38167 (*d* = 0.013771), clustered within GC Ib, and was closely related to T-98 (within the vaccine strains cluster; *d* = 0.06027). Compared with the S-class genomic segments, the σNS of the R18-37308 strain displayed the highest sequence homology to that of R18-38167 strain (98.64%). The σNS aa analysis highlighted a F25Y aa substitution that was shared between our strains (Table S14). However, L82M and V120I were found to be unique to the R18-38167 strain. Of note, the R152H, M257F, T273V and M350T aa substitutions were found only in GC II.

Taking into consideration all the results obtained in this section, a new overall classification of the NC strains could be concluded if the entire segment-based genotypic clusters were included (Table 2). Moreover, based on the closest isolates inferred from phylogenetic proteins of the 12 coding genomic regions, σC-based clustering was only supported by σA, σNS and µNS and partially by µB. This indicates insufficient support for depending on σC for strain genotyping. 

In terms of the genetic similarity between the NC ARVs and the vaccine strain, the S2 segment of R18-37308 and R18-38167 shared the highest nucleotide sequence identity with the vaccine strain S1133 (91.54%; Table 1). In contrast, the L3 segment of the NC isolates displayed the lowest nucleotide sequence homology with the S1133 vaccine strain, of about 74% (Table 1). 

### 3.4. Visualization of the Whole Genome Alignment

**Figure 3:**
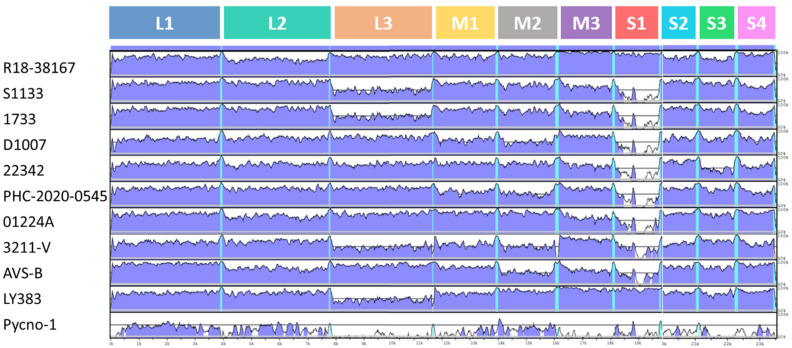
The mVISTA plot for whole genomic nucleotide alignment, showing the alignment results of the R18-37308 strain compared with the R18-38167 strain and 10 other ARV reference strains. The areas in blue denote similarities above or equal to 90%, and the white areas refer to similarities <90%. The scale bar defines the approximate length of the concatenated genome. The cyan-colored lines refer to UTRs flanking the coding regions. Accession numbers of the sequences used for the whole genome alignment are shown in Table S20.

The nucleotide sequence similarity index of individual genome segments of R18-37308 showed less divergence from R18-38167 but a high divergence from all ten ARV reference strains (Figure 3). The genome visualization by mVista further reinforced the phylogenetic findings described above. For example, segment S1 (containing the *σC* gene) showed high nucleotide sequence similarity above 90% for R18-38167 and LY383 with that of R18-37308. Significant nucleotide sequence similarities, greater than 90%, were observed in most regions of the L1, L2, M1, M3, S2, S3 and S4 segments between the newly characterized ARVs and other reference strains. In contrast, R18-37308 and R18-38167 showed low identity (<80%) with the reference ARV genomes in the L3, M2 and S1 segments, with the minimum identity detected at the S1 segment 3′ terminus (i.e., the σC region). Overall, when R18-38167 was compared with ARV reference strains, a substantial genetic relationship was observed with the LY383 strain in all the segments, except for the L3 and M1 segments. The concatenated genome segments revealed that at least six reference strains (01224A, LY383, 1133, 1733, AVS-B and PHC-2020-0545) shared high sequence similarity in some genomic segments with the NC strains. The brown-eared bulbul-origin ARV strain Pynco-1 shared the least sequence similarity with the study strains over the entire genome; thus, there was no reassortment probability. The turkey strain 22342 showed lower sequence identity with R18-37308 and R18-38167 in the S3 genomic segment than the other partridge and chicken reference strains. Most importantly, the S1 segment (encoding the virus attachment protein σC) of R18-37308 and R18-38167 displayed marked variations when compared with the S1133 vaccine strain, suggesting probable failure of the vaccine containing the classical S1133 strain against the NC strains.

### 3.5. Recombination Analysis Indicates the Novelty of the Study Strains

**Figure 4:**
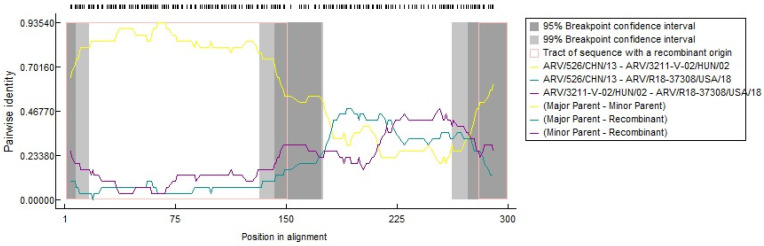
Recombination plot showing the orientation of recombination breakpoints and probable parental strains.

All 12 genes were checked for the incidence of recombination. However, a single algorithm (RDP) detected a single recombination event in the *p10* gene of R18-37308 and recorded two breakpoints at positions 151 and 279 at 95% confidence intervals, giving rise to three intervals (Figure 4). The two intervals at 1-151 and 280–300 are denoted by the 3211-V Hungarian isolate as a minor parental strain, whereas the middle interval at 152–279 is denoted by the Chinese isolate, 526, representing the major parental sequence. Even with the recombination event detection at a high recombination rate of 1.336 × 10^−2^ by RDP, the recombination event is still doubtful, since six out of seven algorithms showed no evidence of recombination. The lack of sufficient evidence for the incidence of recombination negates the possibility that our strains emerged from previously circulating strains and supports the novelty of the present study strains.

## 4. Discussion

ARV-mediated arthritis/tenosynovitis syndrome has been reported in domestic poultry since the 1950s [44,45,46]. The continuous development of the modern poultry business has revealed symptoms other than the classic arthritis symptoms, including immunosuppression [47], malabsorption syndrome [48], runting–stunting syndrome (RSS) [49], enteric disease [50], and respiratory disease [51]. A surge in ARV diagnosis in poultry in the USA since 2011 shows new cases in flocks of broilers, layer chickens and turkeys [28,33,52,53,54,55,56,57,58]. The availability of genomic sequence information could help uncover the ARV strains’ genetic architecture and molecular evolution and to conduct epidemiological studies and design novel diagnostic methods. The σC, the principal antigenic protein triggering the neutralizing antibodies, is commonly sequenced and used for genotyping [59,60]. However, other genes were found to add significant variability to the reovirus genome [18]. For instance, the µB and λC genes were also found to have potential genetic divergence and were proposed to be used together with σC for a better classification system [61]. Moreover, ARV pathogenicity, so far, is not tightly related to the σC-based genotype, or even to its serotype [62]. The vaccine genogroup, for example, was reported to contain reovirus strains of various pathotypes associated with different organ and tissue tropisms, including the highly pathogenic S1133, 1733 and 2408, with several tissue and organ tropisms, the intermediate pathogenic 2035 strain and the non-arthrogenic 2177 strain that lacks tendon tropism [1,63,64]. Furthermore, the S4-encoded protein (σNS) depicted distinct lineages that better define the ARV host origin and was used in diagnostic analysis [65,66,67]. Consequently, single-gene analysis is no longer sufficient to define the genotype and pathotype of an ARV strain. Whole-genome sequencing (WGS), therefore, is essential to define the genetic makeup of ARV strains; of particular interest, those escaping vaccine-mediated immune responses [18,68]. The NGS technologies have revolutionized deep genomic analysis. Currently, NGS is a rapid processing approach in clinical virology that shows significant efficacy and generates tremendous amounts of low-cost data in a reasonably short time [69]. NGS successfully characterized two variant ARVs co-infecting young layer chickens in California [70]. Herein, two NC strains, recovered from broilers showing arthritis symptoms, were whole-genome sequenced for more precise molecular surveillance and to understand their molecular evolution and probable origin. The molecular characterization of ARV isolates is mostly based on sequencing analysis of a partial S1 gene sequence [26] that classifies ARVs into five [28,71] or six [24,26,72] genotypic clusters. In the same manner, our σC-based genotyping showed six GCs. A previous study in Taiwan showed that the divergence among ARVs started after 1986, leading to the emergence of genetic variants grouped within six distinct clusters and the report of the first two ARV GC VI strains in 1992 [55]. Moreover, GC VI was detected for the first time in PA poultry during the period 2012–2014, which involved nine broiler strains and a single turkey strain [24]. Later, a decline in GC I representation along with a GC VI surge was reported in California [18]. For instance, ARVs belonging to GC VI were the second most predominant isolates in California [18]. Our NC strains were entirely clustered within GC VI. This notable shift could be attributed to the use of autogenous vaccines, since a single GC V variant along with two GC I isolates were introduced to breeders that favored the better fitness of GC VI over the others. Moreover, these NC isolates were detected in broilers showing arthritis/tenosynovitis. ARV isolates belonging to GC II, IV, V and VI were found to be associated with tendinitis/tenosynovitis in experimentally infected SPF birds, which further supports our observation [72].

Our phylogenetic analysis of the individual ARV genomic segments resulted in tree topologies different from the σC-based tree topology, and with the majority of strains, including R18-37308 and R18-38167, clustering differently, depending on the gene or segment used for classification. Likewise, a previous phylogenetic analysis of the individual genes of the AVS-B strain displayed no common ancestor and suggested that it has acquired its genetic makeup from diverse strains via sophisticated evolutionary mechanisms, including genomic segment reassortment and subsequent divergence within individual AVS-B genes [73]. The aa analysis of R18-37308 and R18-38167 showed multiple aa substitutions across all genomic segments. Several aa substitutions were reported in the reovirus isolates v-S1133, C78 and GX110116, including A214S, S303A, M530L, K757R and I1193L gene segments in the Lambda A segment and three aa changes involving T262S, T/S390 and N482D in the µB segment that did not appear in the R18-37308 and R18-38167 strains [74]. This could be justified by the fact that these vaccine strains were clustered within GC I for the Lambda A and µB sequences, respectively, whereas our isolates were clustered within GC III in the case of Lambda A and within GC II in case of µB. 

In this study, we also include a deep genetic characterization of the ARV R18-37308 and R18-38167 coding regions at the amino acid level. Previous studies detected a C_2_H_2_ zinc-binding motif (aa 177/182–202/207) in the λA protein of ARVs [53,65,75] that we also detected in the NC isolates. This motif was also described in the mammalian reovirus λ1 (aa 183–203) [76] and the grass carp reovirus VP3 (aa 119–140) [77]. We detected an N179S aa substitution (within the C_2_H_2_ zinc-binding motif) in the NC strains that was previously detected in turkey reovirus strains [75]. On the other hand, the λB polymerase regions responsible for the accurate positioning of template nucleosides (aa 515–529 and 583–588), NTP priming (aa 557–568) and RNA polymerase activity (aa 728–735) were all detected in the NC strains and were found to be conserved and very similar to what has been observed in turkey reoviruses [75]. λC mediates the guanylyl transferase activity in the cap formation of viral mRNA [78,79]. Genomic studies of ARVs observed variations in specific λC regions associated with no pathogenic consequences; these mutations, however, might impact the capping activity and, consequently, the viral replication cycle [18,53,80]. The S-adenosyl-L-methionine (SAM)-binding pocket for methyltransferase was previously defined in the λC protein at aa residues 822–830, and the 169/188K residues required for guanylyl transferase activity were found to be conserved in the NC strains as well as in other chicken strains [75]. Furthermore, the μA of the NC strains contained the 458-LALDPPF-464 motif that resembles the N-6 adenine-specific DNA methylase; however, R18-38167 encountered an L458M aa substitution at this motif [33,81]. We also detected a basic motif (NH2-RSRLKGFQKRP-COOH) at aa residues 101–111 that was also reported in turkey strains; however, the latter strains have an aa substitution at aa 106 that might be host-specific [82]. The major outer capsid protein μB is related to virus stability, membrane association capacity and binding affinity, thereby influencing the replication and infectivity of ARVs [32,83]. We encountered several point mutations along the μB protein that indicate a high molecular divergence. The M2 segment was reported to be the ARV segment that accounts for the most variability among the ARV strains [18,84].

The aa analysis of the S1 segment revealed that the P10 of the R18-37308 and R18-38167 NC strains lacked the first 3 aa residues existent in other ARVs due to a non-synonymous mutation at the start codon, giving rise to 96 aa protein. The common defining biophysical properties of the FAST protein family include their small size feature of 95–140 aa residues [85], similar to our P10. Moreover, the N-terminus of P10 is the fusogenic extracellular domain that is essential for permeabilizing activity, since the fusogenic capacity is abolished upon its deletion, with no impact on virus association with cellular membranes [86]. However, we encountered normal levels of fusogenic activity in the ARVs, as indicated by syncytia formation during the virus propagation in cell culture. This could be attributed to the partial deletion of short aa residues that might not be essential for fusogenic activity rather than the deletion of the entire N-terminus required for this activity. Furthermore, an aa mutation (L2S) at this deleted part was reported in turkey strains without any reported impact [87]. The turkey arthritis/enteric reovirus (TARV/TERV)-predicted transmembrane motif of P10 was detected in our isolates with conserved sequences at only the N-terminus (44-YLAAGGG-50) and several point mutations in the remaining sequence of this motif [87]. Additionally, we detected an L insertion at aa 51 in the P10 of R18-37308 and R18-38167, whereas turkey strains contained a V insertion at the same aa that was also noted in the turkey isolate (22342) included in our analysis. The P17 nuclear localization signal (NLS) motif (NH2-TAAKRSRGVD-COOH) at aa 119–128 was characteristic of the P17-based GC IV, which includes the NC strains, and was different from the chicken vaccine strain and the previously reported NLS motif in turkey strains [87].

σC is a crucial antigenic determinant and is highly prone to molecular divergence, which was reported to be higher at the aa residues 42–173 [88]. Most of the characteristic mutations observed in the σC sequences of the two NC isolates occurred at the same location. In fact, we detected unique aa substitutions at residues 101 and 108 that were characteristic of each of the NC strains. Different substitutions were found at those two residues in several turkey arthritis viruses (TARV-MN6 and TARV-MN5) [87]. In addition, we encountered the T256V mutation in the R18-38167 strain, which is located at the C-terminus of the σC polypeptide chain (residues 156–326), constituting the globular head domain and a slender shaft responsible for receptor binding [89]. Moreover, T256 was found to be exposed on the protein surface [90]; in addition, it was exactly adjacent to a previously reported σC antigenic peptide (257–265 aa) [88], which supports the probability of positive selection by neutralizing antibodies and the resultant mutation.

The σA protein is essential for virus replication and has the ability to bind to dsRNA at the N-terminus [91]. A binding-site mutation hampers its dsRNA binding capacity and, consequently, it loses its ability to enter the nucleus; the lack of a σA protein will, therefore, limit virus replication [92,93]. The two arginine residues at 155 and 273 were found to be significant, and the σA R155/273A mutant lacks the capability to enter the nucleolus, which is associated with a reduction in ATP formation that is required for virus replication [94]. Both 155R and 273R were found to be conserved in the NC strains, indicating optimal performance of the σA protein. 

The σB protein, an outer capsid protein, includes neutralizing epitopes that is group-specific [95]. It contains hydrophilic areas, and the 137–173 aa residues were reported to comprise the largest hydrophilic region [96]. R18-37308 and R18-38167 presented a mutation at aa 144 within this region; however, the polar, uncharged S aa was substituted with another polar, uncharged aa, G. This aa is exposed on the surface, which explains the possibility of a mutation occurring due to positive selection. A conserved CHCC zinc-binding motif (aa 51–75) was found to be conserved in the NC isolates, with a single A65S mutation in R18-37308 as well as in other chicken strains compared with the S1133 strain. In contrast, in a previous study, three substitutions at aa 61, 62 and 64 were identified in turkey strains, which appear to be host-specific [87]. H23C substitution in the CCHC motif of HIV-1 NCp7 caused the entire loss of infectivity [97].

The σNS protein is pivotal for the ssRNA-binding activity, and any changes may be associated with a reduction or an increase in the viral packaging and replication efficacy, thus influencing ARV pathogenicity [98]. The σNS N-terminus (38 aa residues) is required for RNA binding [99]. Although we detected a mutation at aa 25 within the RNA-binding domain of R18-37308 and R18-38167, we suspect that this would not have any effect on virus replication because a previous study reported that a Y25A mutation did not impact σNS activity whereas two positively charged residues (R6 and R29) in the σNS N-terminus were necessary for RNA binding, and these were conserved in our sequences [100]. Moreover, the σNS epitope B (180-MLDMVDGRP-188) was found to be conserved, with a single aa substitution (M183I) in the two NC strains. The same mutation was previously reported in a TERV [87]. In addition, the genomic characterization of ARV from wild birds in South Korea revealed 10 aa substitutions in σNS that were also detected in the σNS sequences of the two NC isolates, except for a single aa substitution at aa 72 [101]. Furthermore, these 10 substitutions were also detected in the Californian isolate K1600657 of chicken origin and were found to be significantly associated with severe clinical signs [18]. 

Finally, our recombination analysis failed to provide strong evidence of any incidence of recombination as six out of seven algorithms did not detect any recombination breakpoints, which assures the lack of any parental strains. A wild bird ARV was found to be recombined with GX110116 (a Chinese vaccine strain) in South Korea at breakpoints ~300 and 500 and with K1600657 (Californian, of chicken origin) at around 700–900 nucleotide sequence positions [101], which disagrees with our lack of recombination events; this is most probably due to spatial and temporal variations as well as differences in the host (wild birds).

## 5. Conclusions

We depicted the whole genome characterization of two ARVs isolated from broilers in NC and found them to be closely related to chicken-origin ARVs. Several amino acid substitutions were detected across the whole genome, which indicate the continuous accumulation of point mutations. The high divergence of the sigma C portion of the S1 segment of the NC isolates from that of the classical vaccine strain may indicate the possible failure of the S1133 strain to control the ARVs’ infection and associated clinical signs that mandate active molecular surveillance programs for any emerging or circulating strains.

## Figures and Tables

**Table 1:** Genomic organization of the current study strains.

Genomic Segment	Length (bp)	ORF Localization		Similarity (%) to S1133 *	
		Start	End	R18-37308	R18-38167
L1	3959	22	2903	88.67	88.68
L2	3830	15	3794	83.46	89.79
L3	3907	13	3870	74.25	74.12
M1	2283	13	2211	88.30	88.04
M2	2158	30	2060	85.14	84.53
M3	1996	25	1932	82.07	82.07
S1	1643	31	321	74.39	75.09
		293	733	80.77	80.58
		630	1610	86.49	86.49
S2	1324	16	1266	91.54	91.54
S3	1202	31	1134	87.27	88.10
S4	1192	24	1127	82.63	82.47

ORF: open reading frame, *: Similarity percentage is based on nucleotide sequence.

**Table 2:** Whole-genome-based genotypic characterization compared with the classical σC genotyping.

Strain	σC-Based Genotype	Whole-Genome-Based Genotyping
R18-37308	VI	L1-III/L2-Id/L3-II/M1-IIIa/M2-II/M3-II/S1-VI/S2-I/S3-Ic/S4-II *
R18-38167	VI	L1-III/L2-II/L3-II/M1-IIIa/M2-II/M3-II/S1-VI/S2-I/S3-Ib/S4-II

* σC was used as a representative of the S1 segment for its vital role and also because of the similar clustering obtained for P10 and P17 compared to that of σC.

## Data Availability

The whole-genome sequences of R18-37308 and R18-38167 were deposited in GenBank with accession numbers OR546351-OR546370. Moreover, the sequences used in this study for the phylogenetic analysis are openly available in the NCBI GenBank repository with the accession numbers mentioned in Tables S3 and S14.

## Supplementary Materials

The following supporting information can be downloaded at: https://www.mdpi.com/article/10.3390/v15112191/s1, Table S1. Long-read sequencing data generated for our NC strains. Table S2. Evolutionary divergence estimates between σC sequences of ARV isolates. Table S3. Accession numbers of sequences used for the phylogenetic analysis of ARV σC for genotyping. Table S4. Reference sequences used for the phylogenetic analysis of genomic amino acid sequences. Table S5. Evolutionary divergence estimates between λA sequences of ARV isolates. Table S6. The characteristic amino acid substitutions in the L-class genomic segments. Table S7. Evolutionary divergence estimates between λB sequences of ARV isolates. Table S8. Evolutionary divergence estimates between λC sequences of ARV isolates. Table S9. Evolutionary divergence estimates between µA sequences of ARV isolates. Table S10. The characteristic amino acid substitutions in the M-class genomic segments. Table S11. Evolutionary divergence estimates between µB sequences of ARV isolates. Table S12. Evolutionary divergence estimates between µNS sequences of ARV isolates. Table S13. Evolutionary divergence estimates between P10 sequences of ARV isolates. Table S14. The characteristic amino acid substitutions in the S-class genomic segments. Table S15. Evolutionary divergence estimates between P17 sequences of ARV isolates. Table S16. Evolutionary divergence estimates between σA sequences of ARV isolates. Table S17. Evolutionary divergence estimates between σB sequences of ARV isolates. Table S18. Evolutionary divergence estimates between σNS sequences of ARV isolates. Table S19. Accession numbers of sequences used for the phylogenetic analysis of ARV coding sequences for genotypic clustering. Table S20. Accession numbers of sequences used for the whole-genome alignment.
