# Supplementary material for: Whole Genomic Constellation of Avian Reovirus Strains Isolated from Broilers with Arthritis in North Carolina, USA
Source: Viruses. 2023 Oct 31;15(11):2191. doi: 10.3390/v15112191 (PMC10675200; doi:10.3390/v15112191)
Supplement: Supplementary file 1 [file viruses-15-02191-s001.zip › viruses-2667692-supplementary.pdf]

Table S1. Long-read sequencing data generated for our NC strains.

Strain R18-37308										
**Segment**	L1	L2	L3	M1	M2	M3	S1	S2	S3	S4
**Mean read length**	1,870	1,421	1,574	1,188	1,052	1,171	964	961	817	860
**Mean read Phred**	13	13	13	12	12	12	12	12	12	12
**Number of reads**	1,751	1,420	1,934	1,070	1,944	1,174	1,052	847	1,353	1,396
**Read length N50**	2,810	2,147	2,511	1,662	1,505	1,648	1,286	1,189	1,005	1,025
**Total bases**	3,273,802	2,017,584	3,044,043	1,271,195	2,045,941	1,374,420	1,014,061	813,606	1,105,732	1,200,578
**Average depth**	827	527	779	557	948	689	617	615	920	1,007

Strain R18-38167										
**Segment**	L1	L2	L3	M1	M2	M3	S1	S2	S3	S4
**Mean read length**	1,665	1,509	1,385	1,230	1,088	1,142	1,182	919	814	842
**Mean read Phred**	12	13	13	12	12	12	12	12	12	12
**Number of reads**	2,011	882	1,526	577	1,410	1,451	1,172	1,511	1,540	1,089
**Read length N50**	2,625	2,259	2,138	1,764	1,540	1,582	1,504	1,153	1,052	1,007
**Total bases**	3,347,240	1,331,138	2,114,000	709,717	1,533,470	1,657,549	1,385,528	1,389,099	1,253,631	916,902
**Average depth**	845	348	541	311	711	830	843	1,049	1,043	769

Table S2. Evolutionary divergence estimates between σC sequences of ARV isolates.

Species 1	Species 2	Dist
SD09-1/Ck/CHN/09	C78/Ck/CHN/13	0.00002232
C-98/Ck/CHN/06	MS01/Ck/CHN/17	0.00002232
157/Wb/KOR/15	205/Wb/KOR/18	0.00002232
878-Bi-05/Ck/HUN/05	875-Bi-05/Ck/HUN/05	0.00002232
V-ARV-SD26/Ck/CHN/20	LY383/Ck/CHN/16	0.00002232
MS01/Ck/CHN/17	T-98/Ck/CHN/06	0.00003348
C-98/Ck/CHN/06	T-98/Ck/CHN/06	0.00003348
48/Wb/KOR/15	205/Wb/KOR/18	0.00004464
48/Wb/KOR/15	157/Wb/KOR/15	0.00004464
GuangxiR1/Ck/CHN/00	1733/Ck/USA/97	0.00288176
C78/Ck/CHN/13	Avs-S1133/Ck/CHN/13	0.00288678
SD09-1/Ck/CHN/09	Avs-S1133/Ck/CHN/13	0.00288678
205/Wb/KOR/18	19/Wb/KOR/15	0.00288756
157/Wb/KOR/15	19/Wb/KOR/15	0.00288756
48/Wb/KOR/15	19/Wb/KOR/15	0.00288756
C78/Ck/CHN/13	YJL/Dk/CHN/05	0.00289838
SD09-1/Ck/CHN/09	YJL/Dk/CHN/05	0.00289838
205/Wb/KOR/18	19/Wb/KOR/18	0.00290353
157/Wb/KOR/15	19/Wb/KOR/18	0.00290353
48/Wb/KOR/15	19/Wb/KOR/18	0.00290353
108/Wb/KOR/15	205/Wb/KOR/18	0.00291016

108/Wb/KOR/15	157/Wb/KOR/15	0.00291016
108/Wb/KOR/15	48/Wb/KOR/15	0.00291016
C78/Ck/CHN/13	YH/Dk/CHN/05	0.00291605
SD09-1/Ck/CHN/09	YH/Dk/CHN/05	0.00291605
C78/Ck/CHN/13	13/Wb/KOR/18	0.00291801
SD09-1/Ck/CHN/09	13/Wb/KOR/18	0.00291801
C78/Ck/CHN/13	SD10-1/Ck/CHN/09	0.00294328
SD09-1/Ck/CHN/09	SD10-1/Ck/CHN/09	0.00294328
C78/Ck/CHN/13	GX/2010/1/Ck/CHN/10	0.00294862
SD09-1/Ck/CHN/09	GX/2010/1/Ck/CHN/10	0.00294862
19/Wb/KOR/15	19/Wb/KOR/18	0.00572412
108/Wb/KOR/15	19/Wb/KOR/18	0.00572440
108/Wb/KOR/15	19/Wb/KOR/15	0.00573075
GuangxiR1/Ck/CHN/00	GuangxiR2/Ck/CHN/00	0.00573456
SD10-1/Ck/CHN/09	GX/2010/1/Ck/CHN/10	0.00573565
Avs-S1133/Ck/CHN/13	YJL/Dk/CHN/05	0.00574052
YH/Dk/CHN/05	13/Wb/KOR/18	0.00574478
YJL/Dk/CHN/05	YH/Dk/CHN/05	0.00574747
YJL/Dk/CHN/05	13/Wb/KOR/18	0.00574943
SD10-1/Ck/CHN/09	13/Wb/KOR/18	0.00574968
GX/2010/1/Ck/CHN/10	13/Wb/KOR/18	0.00575502
Avs-S1133/Ck/CHN/13	YH/Dk/CHN/05	0.00575819
Avs-S1133/Ck/CHN/13	13/Wb/KOR/18	0.00576015
SD10-1/Ck/CHN/09	YH/Dk/CHN/05	0.00577005
SD10-1/Ck/CHN/09	YJL/Dk/CHN/05	0.00577470
GX/2010/1/Ck/CHN/10	YH/Dk/CHN/05	0.00577539
GX/2010/1/Ck/CHN/10	YJL/Dk/CHN/05	0.00578004
SD10-1/Ck/CHN/09	Avs-S1133/Ck/CHN/13	0.00578542
205/Wb/KOR/18	106/Wb/KOR/19	0.00578956
157/Wb/KOR/15	106/Wb/KOR/19	0.00578956
48/Wb/KOR/15	106/Wb/KOR/19	0.00578956
Avs-S1133/Ck/CHN/13	GX/2010/1/Ck/CHN/10	0.00579076
205/Wb/KOR/18	T-98/Ck/CHN/06	0.00582166
157/Wb/KOR/15	T-98/Ck/CHN/06	0.00582166
48/Wb/KOR/15	T-98/Ck/CHN/06	0.00582166
205/Wb/KOR/18	MS01/Ck/CHN/17	0.00583282
205/Wb/KOR/18	C-98/Ck/CHN/06	0.00583282
157/Wb/KOR/15	MS01/Ck/CHN/17	0.00583282
157/Wb/KOR/15	C-98/Ck/CHN/06	0.00583282
48/Wb/KOR/15	MS01/Ck/CHN/17	0.00583282
48/Wb/KOR/15	C-98/Ck/CHN/06	0.00583282
C78/Ck/CHN/13	GX110116/Ck/CHN/11	0.00588046

SD09-1/Ck/CHN/09	GX110116/Ck/CHN/11	0.00588046
01224B/Ck/USA/14	D12/Ck/CAN/14	0.00602304
108/Wb/KOR/15	106/Wb/KOR/19	0.00858811
1733/Ck/USA/97	GuangxiR2/Ck/CHN/00	0.00859399
GuangxiR2/Ck/CHN/00	2408/Ck/USA/83	0.00859751
19/Wb/KOR/18	106/Wb/KOR/19	0.00860380
T-98/Ck/CHN/06	GuangxiR2/Ck/CHN/00	0.00860940
19/Wb/KOR/15	106/Wb/KOR/19	0.00861015
108/Wb/KOR/15	T-98/Ck/CHN/06	0.00862021
MS01/Ck/CHN/17	GuangxiR2/Ck/CHN/00	0.00862056
C-98/Ck/CHN/06	GuangxiR2/Ck/CHN/00	0.00862056
GuangxiR1/Ck/CHN/00	2408/Ck/USA/83	0.00862444
108/Wb/KOR/15	MS01/Ck/CHN/17	0.00863137
108/Wb/KOR/15	C-98/Ck/CHN/06	0.00863137
19/Wb/KOR/18	T-98/Ck/CHN/06	0.00863590
T-98/Ck/CHN/06	GuangxiR1/Ck/CHN/00	0.00863632
19/Wb/KOR/15	T-98/Ck/CHN/06	0.00864225
19/Wb/KOR/18	MS01/Ck/CHN/17	0.00864706
19/Wb/KOR/18	C-98/Ck/CHN/06	0.00864706
MS01/Ck/CHN/17	GuangxiR1/Ck/CHN/00	0.00864748
C-98/Ck/CHN/06	GuangxiR1/Ck/CHN/00	0.00864748
S1133/Ck/CHN/14	GX110058/Ck/CHN/11	0.00864781
GX/2010/1/Ck/CHN/10	GX110116/Ck/CHN/11	0.00865050
19/Wb/KOR/15	MS01/Ck/CHN/17	0.00865341
19/Wb/KOR/15	C-98/Ck/CHN/06	0.00865341
205/Wb/KOR/18	GuangxiR2/Ck/CHN/00	0.00865593
157/Wb/KOR/15	GuangxiR2/Ck/CHN/00	0.00865593
48/Wb/KOR/15	GuangxiR2/Ck/CHN/00	0.00865593
SD10-1/Ck/CHN/09	GX110116/Ck/CHN/11	0.00866749
205/Wb/KOR/18	GuangxiR1/Ck/CHN/00	0.00868286
157/Wb/KOR/15	GuangxiR1/Ck/CHN/00	0.00868286
48/Wb/KOR/15	GuangxiR1/Ck/CHN/00	0.00868286
13/Wb/KOR/18	GX110116/Ck/CHN/11	0.00868686
YH/Dk/CHN/05	GX110116/Ck/CHN/11	0.00870722
YJL/Dk/CHN/05	GX110116/Ck/CHN/11	0.00871187
Avs-S1133/Ck/CHN/13	GX110116/Ck/CHN/11	0.00872259
D12/Ck/CAN/14	D6/Ck/CAN/14	0.00903275
D8/Ck/CAN/14	D12/Ck/CAN/14	0.00903700
108/Wb/KOR/15	GuangxiR2/Ck/CHN/00	0.01145448
T-98/Ck/CHN/06	106/Wb/KOR/19	0.01145496
MS01/Ck/CHN/17	106/Wb/KOR/19	0.01146612
C-98/Ck/CHN/06	106/Wb/KOR/19	0.01146612

19/Wb/KOR/18	GuangxiR2/Ck/CHN/00	0.01147018
19/Wb/KOR/15	GuangxiR2/Ck/CHN/00	0.01147653
T-98/Ck/CHN/06	2408/Ck/USA/83	0.01147696
108/Wb/KOR/15	GuangxiR1/Ck/CHN/00	0.01148141
1733/Ck/USA/97	2408/Ck/USA/83	0.01148387
MS01/Ck/CHN/17	2408/Ck/USA/83	0.01148812
C-98/Ck/CHN/06	2408/Ck/USA/83	0.01148812
T-98/Ck/CHN/06	1733/Ck/USA/97	0.01149576
19/Wb/KOR/18	GuangxiR1/Ck/CHN/00	0.01149710
19/Wb/KOR/15	GuangxiR1/Ck/CHN/00	0.01150345
MS01/Ck/CHN/17	1733/Ck/USA/97	0.01150692
C-98/Ck/CHN/06	1733/Ck/USA/97	0.01150692
205/Wb/KOR/18	2408/Ck/USA/83	0.01152349
157/Wb/KOR/15	2408/Ck/USA/83	0.01152349
48/Wb/KOR/15	2408/Ck/USA/83	0.01152349
205/Wb/KOR/18	1733/Ck/USA/97	0.01154229
157/Wb/KOR/15	1733/Ck/USA/97	0.01154229
48/Wb/KOR/15	1733/Ck/USA/97	0.01154229
205/Wb/KOR/18	113/Wb/KOR/15	0.01157353
157/Wb/KOR/15	113/Wb/KOR/15	0.01157353
48/Wb/KOR/15	113/Wb/KOR/15	0.01157353
D1/Ck/CAN/14	D3/Ck/CAN/14	0.01217911
106/Wb/KOR/19	GuangxiR2/Ck/CHN/00	0.01428924
106/Wb/KOR/19	GuangxiR1/Ck/CHN/00	0.01431616
108/Wb/KOR/15	2408/Ck/USA/83	0.01432204
19/Wb/KOR/18	2408/Ck/USA/83	0.01433774
108/Wb/KOR/15	1733/Ck/USA/97	0.01434084
19/Wb/KOR/15	2408/Ck/USA/83	0.01434409
19/Wb/KOR/18	1733/Ck/USA/97	0.01435654
19/Wb/KOR/15	1733/Ck/USA/97	0.01436289
108/Wb/KOR/15	113/Wb/KOR/15	0.01437208
19/Wb/KOR/18	113/Wb/KOR/15	0.01438778
19/Wb/KOR/15	113/Wb/KOR/15	0.01439413
T-98/Ck/CHN/06	C78/Ck/CHN/13	0.01457470
T-98/Ck/CHN/06	SD09-1/Ck/CHN/09	0.01457470
MS01/Ck/CHN/17	C78/Ck/CHN/13	0.01458586
MS01/Ck/CHN/17	SD09-1/Ck/CHN/09	0.01458586
C-98/Ck/CHN/06	C78/Ck/CHN/13	0.01458586
C-98/Ck/CHN/06	SD09-1/Ck/CHN/09	0.01458586
205/Wb/KOR/18	C78/Ck/CHN/13	0.01464356
205/Wb/KOR/18	SD09-1/Ck/CHN/09	0.01464356
157/Wb/KOR/15	C78/Ck/CHN/13	0.01464356

157/Wb/KOR/15	SD09-1/Ck/CHN/09	0.01464356
48/Wb/KOR/15	C78/Ck/CHN/13	0.01464356
48/Wb/KOR/15	SD09-1/Ck/CHN/09	0.01464356
D8/Ck/CAN/14	01224B/Ck/USA/14	0.01501539
01224B/Ck/USA/14	D6/Ck/CAN/14	0.01503346
**D12/Ck/CAN/14**	**R18-38167/Ck/USA/18**	**0.01509024**
106/Wb/KOR/19	2408/Ck/USA/83	0.01715680
106/Wb/KOR/19	1733/Ck/USA/97	0.01717560
T-98/Ck/CHN/06	71/Wb/KOR/15	0.01722464
106/Wb/KOR/19	113/Wb/KOR/15	0.01722916
T-98/Ck/CHN/06	S1133/Ck/CHN/14	0.01723175
MS01/Ck/CHN/17	71/Wb/KOR/15	0.01723580
C-98/Ck/CHN/06	71/Wb/KOR/15	0.01723580
71/Wb/KOR/15	S1133/Ck/CHN/14	0.01724285
MS01/Ck/CHN/17	S1133/Ck/CHN/14	0.01724291
C-98/Ck/CHN/06	S1133/Ck/CHN/14	0.01724291
T-98/Ck/CHN/06	113/Wb/KOR/15	0.01726126
MS01/Ck/CHN/17	113/Wb/KOR/15	0.01727242
C-98/Ck/CHN/06	113/Wb/KOR/15	0.01727242
205/Wb/KOR/18	71/Wb/KOR/15	0.01731582
157/Wb/KOR/15	71/Wb/KOR/15	0.01731582
48/Wb/KOR/15	71/Wb/KOR/15	0.01731582
205/Wb/KOR/18	S1133/Ck/CHN/14	0.01732292
157/Wb/KOR/15	S1133/Ck/CHN/14	0.01732292
48/Wb/KOR/15	S1133/Ck/CHN/14	0.01732292
T-98/Ck/CHN/06	GX/2010/1/Ck/CHN/10	0.01734475
MS01/Ck/CHN/17	GX/2010/1/Ck/CHN/10	0.01735591
C-98/Ck/CHN/06	GX/2010/1/Ck/CHN/10	0.01735591
T-98/Ck/CHN/06	SD10-1/Ck/CHN/09	0.01736173
MS01/Ck/CHN/17	SD10-1/Ck/CHN/09	0.01737289
C-98/Ck/CHN/06	SD10-1/Ck/CHN/09	0.01737289
T-98/Ck/CHN/06	13/Wb/KOR/18	0.01738110
MS01/Ck/CHN/17	13/Wb/KOR/18	0.01739226
C-98/Ck/CHN/06	13/Wb/KOR/18	0.01739226
T-98/Ck/CHN/06	YH/Dk/CHN/05	0.01740147
T-98/Ck/CHN/06	YJL/Dk/CHN/05	0.01740612
MS01/Ck/CHN/17	YH/Dk/CHN/05	0.01741263
C-98/Ck/CHN/06	YH/Dk/CHN/05	0.01741263
205/Wb/KOR/18	GX/2010/1/Ck/CHN/10	0.01741360
157/Wb/KOR/15	GX/2010/1/Ck/CHN/10	0.01741360
48/Wb/KOR/15	GX/2010/1/Ck/CHN/10	0.01741360
T-98/Ck/CHN/06	Avs-S1133/Ck/CHN/13	0.01741684

MS01/Ck/CHN/17	YJL/Dk/CHN/05	0.01741728
C-98/Ck/CHN/06	YJL/Dk/CHN/05	0.01741728
MS01/Ck/CHN/17	Avs-S1133/Ck/CHN/13	0.01742800
C-98/Ck/CHN/06	Avs-S1133/Ck/CHN/13	0.01742800
205/Wb/KOR/18	SD10-1/Ck/CHN/09	0.01743059
157/Wb/KOR/15	SD10-1/Ck/CHN/09	0.01743059
48/Wb/KOR/15	SD10-1/Ck/CHN/09	0.01743059
GuangxiR2/Ck/CHN/00	C78/Ck/CHN/13	0.01743130
GuangxiR2/Ck/CHN/00	SD09-1/Ck/CHN/09	0.01743130
108/Wb/KOR/15	C78/Ck/CHN/13	0.01744211
108/Wb/KOR/15	SD09-1/Ck/CHN/09	0.01744211
205/Wb/KOR/18	13/Wb/KOR/18	0.01744996
157/Wb/KOR/15	13/Wb/KOR/18	0.01744996
48/Wb/KOR/15	13/Wb/KOR/18	0.01744996
19/Wb/KOR/18	C78/Ck/CHN/13	0.01745780
19/Wb/KOR/18	SD09-1/Ck/CHN/09	0.01745780
GuangxiR1/Ck/CHN/00	C78/Ck/CHN/13	0.01745822
GuangxiR1/Ck/CHN/00	SD09-1/Ck/CHN/09	0.01745822
19/Wb/KOR/15	C78/Ck/CHN/13	0.01746415
19/Wb/KOR/15	SD09-1/Ck/CHN/09	0.01746415
205/Wb/KOR/18	YH/Dk/CHN/05	0.01747033
157/Wb/KOR/15	YH/Dk/CHN/05	0.01747033
48/Wb/KOR/15	YH/Dk/CHN/05	0.01747033
205/Wb/KOR/18	YJL/Dk/CHN/05	0.01747497
157/Wb/KOR/15	YJL/Dk/CHN/05	0.01747497
48/Wb/KOR/15	YJL/Dk/CHN/05	0.01747497
205/Wb/KOR/18	Avs-S1133/Ck/CHN/13	0.01748569
157/Wb/KOR/15	Avs-S1133/Ck/CHN/13	0.01748569
48/Wb/KOR/15	Avs-S1133/Ck/CHN/13	0.01748569
D8/Ck/CAN/14	D6/Ck/CAN/14	0.01804743
NLI12-96M/Ck/NLD/01	17203-M/Ck/HUN/06	0.01869933
113/Wb/KOR/15	GuangxiR2/Ck/CHN/00	0.02009554
GuangxiR2/Ck/CHN/00	71/Wb/KOR/15	0.02010356
GuangxiR2/Ck/CHN/00	S1133/Ck/CHN/14	0.02011067
108/Wb/KOR/15	71/Wb/KOR/15	0.02011437
108/Wb/KOR/15	S1133/Ck/CHN/14	0.02012147
113/Wb/KOR/15	GuangxiR1/Ck/CHN/00	0.02012246
19/Wb/KOR/18	71/Wb/KOR/15	0.02013006
GuangxiR1/Ck/CHN/00	71/Wb/KOR/15	0.02013049
T-98/Ck/CHN/06	GX110058/Ck/CHN/11	0.02013182
19/Wb/KOR/15	71/Wb/KOR/15	0.02013641
19/Wb/KOR/18	S1133/Ck/CHN/14	0.02013717

GuangxiR1/Ck/CHN/00	S1133/Ck/CHN/14	0.02013759
71/Wb/KOR/15	GX110058/Ck/CHN/11	0.02014293
MS01/Ck/CHN/17	GX110058/Ck/CHN/11	0.02014298
C-98/Ck/CHN/06	GX110058/Ck/CHN/11	0.02014298
19/Wb/KOR/15	S1133/Ck/CHN/14	0.02014352
GuangxiR2/Ck/CHN/00	GX/2010/1/Ck/CHN/10	0.02020135
108/Wb/KOR/15	GX/2010/1/Ck/CHN/10	0.02021215
GuangxiR2/Ck/CHN/00	SD10-1/Ck/CHN/09	0.02021833
205/Wb/KOR/18	GX110058/Ck/CHN/11	0.02022300
157/Wb/KOR/15	GX110058/Ck/CHN/11	0.02022300
48/Wb/KOR/15	GX110058/Ck/CHN/11	0.02022300
19/Wb/KOR/18	GX/2010/1/Ck/CHN/10	0.02022785
GuangxiR1/Ck/CHN/00	GX/2010/1/Ck/CHN/10	0.02022827
108/Wb/KOR/15	SD10-1/Ck/CHN/09	0.02022914
19/Wb/KOR/15	GX/2010/1/Ck/CHN/10	0.02023419
GuangxiR2/Ck/CHN/00	13/Wb/KOR/18	0.02023770
19/Wb/KOR/18	SD10-1/Ck/CHN/09	0.02024483
GuangxiR1/Ck/CHN/00	SD10-1/Ck/CHN/09	0.02024525
108/Wb/KOR/15	13/Wb/KOR/18	0.02024851
19/Wb/KOR/15	SD10-1/Ck/CHN/09	0.02025118
T-98/Ck/CHN/06	GX110116/Ck/CHN/11	0.02025426
GuangxiR2/Ck/CHN/00	YH/Dk/CHN/05	0.02025807
GuangxiR2/Ck/CHN/00	YJL/Dk/CHN/05	0.02026272
19/Wb/KOR/18	13/Wb/KOR/18	0.02026420
GuangxiR1/Ck/CHN/00	13/Wb/KOR/18	0.02026462
MS01/Ck/CHN/17	GX110116/Ck/CHN/11	0.02026542
C-98/Ck/CHN/06	GX110116/Ck/CHN/11	0.02026542
108/Wb/KOR/15	YH/Dk/CHN/05	0.02026888
19/Wb/KOR/15	13/Wb/KOR/18	0.02027055
GuangxiR2/Ck/CHN/00	Avs-S1133/Ck/CHN/13	0.02027344
108/Wb/KOR/15	YJL/Dk/CHN/05	0.02027352
106/Wb/KOR/19	C78/Ck/CHN/13	0.02027686
106/Wb/KOR/19	SD09-1/Ck/CHN/09	0.02027686
108/Wb/KOR/15	Avs-S1133/Ck/CHN/13	0.02028424
19/Wb/KOR/18	YH/Dk/CHN/05	0.02028457
GuangxiR1/Ck/CHN/00	YH/Dk/CHN/05	0.02028499
19/Wb/KOR/18	YJL/Dk/CHN/05	0.02028922
GuangxiR1/Ck/CHN/00	YJL/Dk/CHN/05	0.02028964
19/Wb/KOR/15	YH/Dk/CHN/05	0.02029092
19/Wb/KOR/15	YJL/Dk/CHN/05	0.02029557
2408/Ck/USA/83	C78/Ck/CHN/13	0.02029886
2408/Ck/USA/83	SD09-1/Ck/CHN/09	0.02029886

19/Wb/KOR/18	Avs-S1133/Ck/CHN/13	0.02029994
GuangxiR1/Ck/CHN/00	Avs-S1133/Ck/CHN/13	0.02030036
19/Wb/KOR/15	Avs-S1133/Ck/CHN/13	0.02030629
1733/Ck/USA/97	C78/Ck/CHN/13	0.02031766
1733/Ck/USA/97	SD09-1/Ck/CHN/09	0.02031766
205/Wb/KOR/18	GX110116/Ck/CHN/11	0.02032312
157/Wb/KOR/15	GX110116/Ck/CHN/11	0.02032312
48/Wb/KOR/15	GX110116/Ck/CHN/11	0.02032312
01224B/Ck/USA/14	R18-38167/Ck/USA/18	0.02104631
D1/Ck/CAN/14	D12/Ck/CAN/14	0.02140594
106/Wb/KOR/19	71/Wb/KOR/15	0.02294913
106/Wb/KOR/19	S1133/Ck/CHN/14	0.02295623
113/Wb/KOR/15	2408/Ck/USA/83	0.02296310
2408/Ck/USA/83	71/Wb/KOR/15	0.02297112
2408/Ck/USA/83	S1133/Ck/CHN/14	0.02297823
113/Wb/KOR/15	1733/Ck/USA/97	0.02298190
1733/Ck/USA/97	71/Wb/KOR/15	0.02298992
1733/Ck/USA/97	S1133/Ck/CHN/14	0.02299703
GuangxiR2/Ck/CHN/00	GX110058/Ck/CHN/11	0.02301074
108/Wb/KOR/15	GX110058/Ck/CHN/11	0.02302155
19/Wb/KOR/18	GX110058/Ck/CHN/11	0.02303724
GuangxiR1/Ck/CHN/00	GX110058/Ck/CHN/11	0.02303766
19/Wb/KOR/15	GX110058/Ck/CHN/11	0.02304359
106/Wb/KOR/19	GX/2010/1/Ck/CHN/10	0.02304691
106/Wb/KOR/19	SD10-1/Ck/CHN/09	0.02306389
2408/Ck/USA/83	GX/2010/1/Ck/CHN/10	0.02306891
106/Wb/KOR/19	13/Wb/KOR/18	0.02308326
2408/Ck/USA/83	SD10-1/Ck/CHN/09	0.02308589
1733/Ck/USA/97	GX/2010/1/Ck/CHN/10	0.02308771
106/Wb/KOR/19	YH/Dk/CHN/05	0.02310363
1733/Ck/USA/97	SD10-1/Ck/CHN/09	0.02310469
2408/Ck/USA/83	13/Wb/KOR/18	0.02310526
106/Wb/KOR/19	YJL/Dk/CHN/05	0.02310828
GuangxiR2/Ck/CHN/00	GX110116/Ck/CHN/11	0.02311086
106/Wb/KOR/19	Avs-S1133/Ck/CHN/13	0.02311900
108/Wb/KOR/15	GX110116/Ck/CHN/11	0.02312167
1733/Ck/USA/97	13/Wb/KOR/18	0.02312406
2408/Ck/USA/83	YH/Dk/CHN/05	0.02312563
2408/Ck/USA/83	YJL/Dk/CHN/05	0.02313028
19/Wb/KOR/18	GX110116/Ck/CHN/11	0.02313736
GuangxiR1/Ck/CHN/00	GX110116/Ck/CHN/11	0.02313778
2408/Ck/USA/83	Avs-S1133/Ck/CHN/13	0.02314100

19/Wb/KOR/15	GX110116/Ck/CHN/11	0.02314371
1733/Ck/USA/97	YH/Dk/CHN/05	0.02314443
1733/Ck/USA/97	YJL/Dk/CHN/05	0.02314908
1733/Ck/USA/97	Avs-S1133/Ck/CHN/13	0.02315980
D8/Ck/CAN/14	R18-38167/Ck/USA/18	0.02408260
R18-38167/Ck/USA/18	D6/Ck/CAN/14	0.02410067
106/Wb/KOR/19	GX110058/Ck/CHN/11	0.0258563
2408/Ck/USA/83	GX110058/Ck/CHN/11	0.0258783
1733/Ck/USA/97	GX110058/Ck/CHN/11	0.0258971
106/Wb/KOR/19	GX110116/Ck/CHN/11	0.02595642
2408/Ck/USA/83	GX110116/Ck/CHN/11	0.02597842
1733/Ck/USA/97	GX110116/Ck/CHN/11	0.02599722
71/Wb/KOR/15	C78/Ck/CHN/13	0.02606887
71/Wb/KOR/15	SD09-1/Ck/CHN/09	0.02606887
S1133/Ck/CHN/14	C78/Ck/CHN/13	0.02607597
S1133/Ck/CHN/14	SD09-1/Ck/CHN/09	0.02607597
113/Wb/KOR/15	C78/Ck/CHN/13	0.02608316
113/Wb/KOR/15	SD09-1/Ck/CHN/09	0.02608316
D1/Ck/CAN/14	01224B/Ck/USA/14	0.02736201
113/Wb/KOR/15	71/Wb/KOR/15	0.02875542
113/Wb/KOR/15	S1133/Ck/CHN/14	0.02876253
71/Wb/KOR/15	GX/2010/1/Ck/CHN/10	0.02883891
S1133/Ck/CHN/14	GX/2010/1/Ck/CHN/10	0.02884602
113/Wb/KOR/15	GX/2010/1/Ck/CHN/10	0.02885321
71/Wb/KOR/15	SD10-1/Ck/CHN/09	0.0288559
S1133/Ck/CHN/14	SD10-1/Ck/CHN/09	0.028863
113/Wb/KOR/15	SD10-1/Ck/CHN/09	0.02887019
71/Wb/KOR/15	13/Wb/KOR/18	0.02887527
S1133/Ck/CHN/14	13/Wb/KOR/18	0.02888237
113/Wb/KOR/15	13/Wb/KOR/18	0.02888956
71/Wb/KOR/15	YH/Dk/CHN/05	0.02889563
71/Wb/KOR/15	YJL/Dk/CHN/05	0.02890028
S1133/Ck/CHN/14	YH/Dk/CHN/05	0.02890274
S1133/Ck/CHN/14	YJL/Dk/CHN/05	0.02890739
113/Wb/KOR/15	YH/Dk/CHN/05	0.02890993
71/Wb/KOR/15	Avs-S1133/Ck/CHN/13	0.028911
113/Wb/KOR/15	YJL/Dk/CHN/05	0.02891458
S1133/Ck/CHN/14	Avs-S1133/Ck/CHN/13	0.02891811
113/Wb/KOR/15	Avs-S1133/Ck/CHN/13	0.0289253
GX110058/Ck/CHN/11	C78/Ck/CHN/13	0.02897604
GX110058/Ck/CHN/11	SD09-1/Ck/CHN/09	0.02897604
D8/Ck/CAN/14	D1/Ck/CAN/14	0.03039829

D1/Ck/CAN/14	D6/Ck/CAN/14	0.03041636
113/Wb/KOR/15	GX110058/Ck/CHN/11	0.0316626
GX110058/Ck/CHN/11	GX/2010/1/Ck/CHN/10	0.03174609
71/Wb/KOR/15	GX110116/Ck/CHN/11	0.03174842
S1133/Ck/CHN/14	GX110116/Ck/CHN/11	0.03175553
113/Wb/KOR/15	GX110116/Ck/CHN/11	0.03176272
GX110058/Ck/CHN/11	SD10-1/Ck/CHN/09	0.03176307
GX110058/Ck/CHN/11	13/Wb/KOR/18	0.03178244
GX110058/Ck/CHN/11	YH/Dk/CHN/05	0.03180281
GX110058/Ck/CHN/11	YJL/Dk/CHN/05	0.03180746
GX110058/Ck/CHN/11	Avs-S1133/Ck/CHN/13	0.03181818
D12/Ck/CAN/14	D3/Ck/CAN/14	0.03354041
GX110058/Ck/CHN/11	GX110116/Ck/CHN/11	0.0346556
D1/Ck/CAN/14	R18-38167/Ck/USA/18	0.03640689
918/Ck/CHN/00	1017-1/Ck/TAI/92	0.0371719
01224B/Ck/USA/14	D3/Ck/CAN/14	0.03949648
D4/Ck/CAN/14	D11/Ck/CAN/14	0.04096062
875-Bi-05/Ck/HUN/05	27614/Ck/USA/13	0.04109293
878-Bi-05/Ck/HUN/05	27614/Ck/USA/13	0.04109293
D8/Ck/CAN/14	D3/Ck/CAN/14	0.04253277
D6/Ck/CAN/14	D3/Ck/CAN/14	0.04255083
D12/Ck/CAN/14	LY383/Ck/CHN/16	0.04297772
D12/Ck/CAN/14	V-ARV-SD26/Ck/CHN/20	0.04297772
R18-38167/Ck/USA/18	D3/Ck/CAN/14	0.04854136
01224B/Ck/USA/14	LY383/Ck/CHN/16	0.0489338
01224B/Ck/USA/14	V-ARV-SD26/Ck/CHN/20	0.0489338
875-Bi-05/Ck/HUN/05	K1600600/Ck/USA/16	0.05116062
878-Bi-05/Ck/HUN/05	K1600600/Ck/USA/16	0.05116062
D8/Ck/CAN/14	LY383/Ck/CHN/16	0.05197008
D8/Ck/CAN/14	V-ARV-SD26/Ck/CHN/20	0.05197008
D6/Ck/CAN/14	LY383/Ck/CHN/16	0.05198815
D6/Ck/CAN/14	V-ARV-SD26/Ck/CHN/20	0.05198815
**D12/Ck/CAN/14**	**R18-37308/Ck/USA/18**	**0.0563058**
R18-38167/Ck/USA/18	LY383/Ck/CHN/16	0.05797868
R18-38167/Ck/USA/18	V-ARV-SD26/Ck/CHN/20	0.05797868
01224B/Ck/USA/14	R18-37308/Ck/USA/18	0.06226187
D1/Ck/CAN/14	LY383/Ck/CHN/16	0.06427205
D1/Ck/CAN/14	V-ARV-SD26/Ck/CHN/20	0.06427205
D8/Ck/CAN/14	R18-37308/Ck/USA/18	0.06529816
D6/Ck/CAN/14	R18-37308/Ck/USA/18	0.06531622
R18-38167/Ck/USA/18	R18-37308/Ck/USA/18	0.07130675
27614/Ck/USA/13	K1600600/Ck/USA/16	0.0717574

875-Bi-05/Ck/HUN/05	T1502036/Ck/USA/15	0.07384464
878-Bi-05/Ck/HUN/05	T1502036/Ck/USA/15	0.07384464
D3/Ck/CAN/14	LY383/Ck/CHN/16	0.07640652
D3/Ck/CAN/14	V-ARV-SD26/Ck/CHN/20	0.07640652
D1/Ck/CAN/14	R18-37308/Ck/USA/18	0.07760013
P3/Cro/JAP/12	PHC-2020-0545/Ck/CHN/20	0.07832624
875-Bi-05/Ck/HUN/05	115940/Ck/USA/16	0.07857908
878-Bi-05/Ck/HUN/05	115940/Ck/USA/16	0.07857908
R18-37308/Ck/USA/18	LY383/Ck/CHN/16	0.0790377
R18-37308/Ck/USA/18	V-ARV-SD26/Ck/CHN/20	0.0790377
D3/Ck/CAN/14	R18-37308/Ck/USA/18	0.0897346
27614/Ck/USA/13	T1502036/Ck/USA/15	0.09444142
918/Ck/CHN/00	3211-V/Ck/HUN/02	0.0952719
27614/Ck/USA/13	115940/Ck/USA/16	0.09917586
T1502036/Ck/USA/15	115940/Ck/USA/16	0.10087041
1017-1/Ck/TAI/92	3211-V/Ck/HUN/02	0.10271708

The strains displaying the closest evolutionary distance to our isolates, are shown in bold red. The evolutionary distance is shown up to d = 0.10271708.

Table S3. Accession numbers of sequences used for phylogenetic analysis of ARV σC for genotyping.

Accession No.	Isolate	Host	Country	Year	Abbreviated name
AB914766	Pycno-1	Brown-eared Bulbul	Japan	2014	Pycno-1/Bul/JAP/14
KX398328	17227-M-10	Chicken	Hungary	2010	17227-M/Ck/HUN/10
KF741752	GX110116	Chicken	China	2011	GX110116/Ck/CHN/11
MW357869	13	wild bird	Korea	2018	13/Wb/KOR/18
DQ198854	YH	Muscovy duck	China	2005	YH/Dk/CHN/05
DQ191363	YJL	Muscovy duck	China	2005	YJL/Dk/CHN/05
KJ476705	GX/2010/1	Chicken	China	2010	GX/2010/1/Ck/CHN/10
KF741772	Avs-S1133	Chicken	China	2013	Avs-S1133/Ck/CHN/13
KP288863	SD10-1	Chicken	China	2009	SD10-1/Ck/CHN/09
KF741722	C78	Chicken	China	2013	C78/Ck/CHN/13
KP288853	SD09-1	Chicken	China	2009	SD09-1/Ck/CHN/09
KF741742	GX110058	Chicken	China	2011	GX110058/Ck/CHN/11
KF741762	S1133	Chicken	China	2014	S1133/Ck/CHN/14
MW357865	71	wild bird	Korea	2015	71/Wb/KOR/15
AF204945	2408	Chicken	USA	1983	2408/Ck/USA/83
KF741732	GuangxiR2	Chicken	China	2000	GuangxiR2/Ck/CHN/00
KF741712	1733	Chicken	USA	1997	1733/Ck/USA/97
KC183744	GuangxiR1	Chicken	China	2000	GuangxiR1/Ck/CHN/00
MW357867	113	wild bird	Korea	2015	113/Wb/KOR/15
MW357872	106	wild bird	Korea	2019	106/Wb/KOR/19
EF057398	T-98	Chicken	China	2006	T-98/Ck/CHN/06
KY860636	MS01	Chicken	China	2017	MS01/Ck/CHN/17
EF057397	C-98	Chicken	China	2006	C-98/Ck/CHN/06
MW357870	19	wild bird	Korea	2018	19/Wb/KOR/18
MW357863	19	wild bird	Korea	2015	19/Wb/KOR/15
MW357871	205	wild bird	Korea	2018	205/Wb/KOR/18
MW357868	157	wild bird	Korea	2015	157/Wb/KOR/15
MW357864	48	wild bird	Korea	2015	48/Wb/KOR/15
MW357866	108	wild bird	Korea	2015	108/Wb/KOR/15
KF741702	526	Chicken	China	2013	526/Ck/CHN/13
MW002453	Reo/Ck/TX/115940	Chicken	USA	2016	115940/Ck/USA/16
MK616649	T1502036	Chicken	USA	2015	T1502036/Ck/USA/15
MK416139	K1600600	Chicken	USA	2016	K1600600/Ck/USA/16
KU169294	Reo/PA/Layer/27614/13	Layer Chicken	USA	2013	27614/Ck/USA/13
KX398248	875-Bi-05	Chicken	Hungary	2005	875-Bi-05/Ck/HUN/05
KX398258	878-Bi-05	Chicken	Hungary	2005	878-Bi-05/Ck/HUN/05
MN879660	D7	Chicken	Canada	2014	D7/Ck/CAN/14
KP173689	Reo/PA/Turkey/22342/13	Turkey	USA	2013	22342/Tk/USA/13
KR476804	D1007	Partridge	Hungary	2008	D1007/PRT/HUN/08
MF686704	K738/14	Chicken	Korea	2014	K738/Ck/KOR/14
MW174790	PHC-2020-0545	Chicken	China	2020	PHC-2020-0545/Ck/CHN/20
LC164026	P3	Crow	Japan	2012	P3/Cro/JAP/12
MN879640	D5	Chicken	Canada	2014	D5/Ck/CAN/14
AF297214	916	Chicken	Taiwan	1992	916/Ck/TAI/92
KX398288	3457-M-11	Chicken	Hungary	2011	3457-M/Ck/HUN/11
KC865792	T1781	Chicken	Hungary	2012	T1781/Ck/HUN/12
KT428304	Reo/PA/Layer/01224A/14	Layer chicken	USA	2014	01224A/Ck/USA/14
OK077999	AHZJ19	Chicken	China	2019	AHZJ19/Ck/CHN/19

MN879690	D10	Chicken	Canada	2014	D10/Ck/CAN/14
KX398278	3211-V-02	Chicken	Hungary	2002	3211-V/Ck/HUN/02
AF297216	1017-1	Chicken	Taiwan	1992	1017-1/Ck/TAI/92
AF297215	918	Chicken	China	2000	918/Ck/CHN/00
MW394462	SDYT2020	Chicken	China	2020	SDYT2020/Ck/CHN/20
NC_015132	AVS-B	Chicken	USA	2006	AVS-B/Ck/USA/06
MK583337	K1600657	Chicken	USA	2016	K1600657/Ck/USA/16
MN879700	D11	Chicken	Canada	2014	D11/Ck/CAN/14
MN879630	D4	Chicken	Canada	2014	D4/Ck/CAN/14
KX398318	17203-M-06	Chicken	Hungary	2006	17203-M/Ck/HUN/06
AF354230	NLI12 96M	Chicken	Netherlands	2001	NLI12-96M/Ck/NLD/01
MN879680	D9	Chicken	Canada	2013	D9/Ck/CAN/13
MF183217	LY383	Chicken	China	2016	LY383/Ck/CHN/16
MW244848	V-ARV-SD26	Chicken	China	2020	V-ARV-SD26/Ck/CHN/20
OR546357	R18-37308	Broiler Chicken	USA	2018	R18-37308/Ck/USA/18
MN879620	D3	Chicken	Canada	2014	D3/Ck/CAN/14
MN879650	D6	Chicken	Canada	2014	D6/Ck/CAN/14
OR546367	R18-38167	Broiler Chicken	USA	2018	R18-38167/Ck/USA/18
MN879710	D12	Chicken	Canada	2014	D12/Ck/CAN/14
KT428314	Reo/PA/Layer/01224B/14	Layer Chicken	USA	2014	01224B/Ck/USA/14
MN879600	D1	Chicken	Canada	2014	D1/Ck/CAN/14
MN879670	D8	Chicken	Canada	2014	D8/Ck/CAN/14
JX478256	091	Duck	China	2009	091/Dk/CHN/09

*Abbreviated name followed the order of Isolate_name/Host/Country_of_origin/Collection_year.

Table S4. Reference sequences used for phylogenetic analysis of genomic amino acid sequences.

Reference strain	σC based genotype	Included/excluded for phylogenetic analysis of genomic ORFs											
		λA	λB	λC	μA	μB	μNS	S1 segment			σA	σB	σNS
								P10	P17	σC			
S1133	I	√	√	√	√	√	√	√	√	√	√	√	√
1733	I	√	√	√	√	√	√	√	√	√	√	√	√
C78	I	√	√	√	√	√	√	√	√	√	√	√	√
T-98	I	√	√	√	√	√	√	√	√	√	√	√	√
MS01	I	√	√	√	√	√	√	√	√	√	√	√	√
526	I	√	√	√	√	√	√	√	√	√	√	√	√
22342	II	√	√	√	√	√	√	√	√	√	√	√	√
PHC-2020-0545	II	√	√	√	√	√	√	√	√	√	√	√	√
D1007	II	√	x[Ұ]	x[Ұ]	√	√	√	√	√	√	√	√	√
01224A	III	√	√	√	√	√	√	√	√	√	√	√	√
AHZJ19	III	√	√	√	√	√	√	√	√	√	√	√	√
3211-V	IV	√	√	√	√	√	√	√	√	√	√	√	√
SDYT2020	V	√	√	√	√	√	√	√	√	√	√	√	√
K1600657	V	√	√	√	√	√	√	√	x[‡]	√	x[Δ]	√	√
LY383	VI	√	√	√	√	√	√	√	√	√	√	√	√
V-ARV-SD26	VI	√	√	√	√	√	√	√	√	√	√	√	√
Pycno-1	Outgroup 1	x[Ұ]	√	√	x[Ұ]	√	√	√	√	√	√	√	√
091	Outgroup 2*	√	√	√	√	√	√	√	x[Ұ]	√	√	√	√

*091 strain (a duck ARV strain) that was found clustered in a separate cluster according to σC (cluster VII), however was also proposed to be used as outgroup in case of the Pycno-1 (bulbul ARV strain) which is extremely evolutionary diverse and masking variations among other strains tested. [‡]: This strain sequence contains degenerate nucleotides that impacted negatively the obtained amino acid sequence. [Δ]: σA sequence of this isolate is incomplete.
√ denotes included in analysis of this genomic segment, whereas x refers to exclusion of this isolate from phylogenetic analysis of this protein sequence. [Ұ]: refers to highly diverse sequence masking evolutionary variations between sequences.

Table S5. Evolutionary divergence estimates between λA sequences of ARV isolates.

Species 1	Species 2	Dist
LY383/Ck/CHN/16	V-ARV-SD26/Ck/CHN/20	0.00003289
1733/Ck/USA/97	C78/Ck/CHN/13	0.00081168
MS01/Ck/CHN/19	T-98/Ck/CHN/06	0.00159225
S1133/Ck/CHN/14	1733/Ck/USA/97	0.00160221
S1133/Ck/CHN/14	C78/Ck/CHN/13	0.00237003
1733/Ck/USA/97	MS01/Ck/CHN/19	0.00318881
PHC-2020-0545/Ck/CHN/20	LY383/Ck/CHN/16	0.00323709
PHC-2020-0545/Ck/CHN/20	V-ARV-SD26/Ck/CHN/20	0.00324805
MS01/Ck/CHN/19	C78/Ck/CHN/13	0.00397855
1733/Ck/USA/97	T-98/Ck/CHN/06	0.00475912
S1133/Ck/CHN/14	MS01/Ck/CHN/19	0.00476909
SDYT2020/Ck/CHN/20	AHZJ19/Ck/CHN/19	0.00478130
C78/Ck/CHN/13	T-98/Ck/CHN/06	0.00554887
S1133/Ck/CHN/14	T-98/Ck/CHN/06	0.00633941
PHC-2020-0545/Ck/CHN/20	SDYT2020/Ck/CHN/20	0.01195701
PHC-2020-0545/Ck/CHN/20	AHZJ19/Ck/CHN/19	0.01222139
526/Ck/CHN/13	K1600657/Ck/USA/16	0.01501246
LY383/Ck/CHN/16	SDYT2020/Ck/CHN/20	0.01517217
V-ARV-SD26/Ck/CHN/20	SDYT2020/Ck/CHN/20	0.01518313
LY383/Ck/CHN/16	AHZJ19/Ck/CHN/19	0.01543655
V-ARV-SD26/Ck/CHN/20	AHZJ19/Ck/CHN/19	0.01544752
1733/Ck/USA/97	526/Ck/CHN/13	0.01636565
MS01/Ck/CHN/19	526/Ck/CHN/13	0.01638902
C78/Ck/CHN/13	526/Ck/CHN/13	0.01715539
1733/Ck/USA/97	SDYT2020/Ck/CHN/20	0.01759093
MS01/Ck/CHN/19	SDYT2020/Ck/CHN/20	0.01761430
1733/Ck/USA/97	AHZJ19/Ck/CHN/19	0.01785532
MS01/Ck/CHN/19	AHZJ19/Ck/CHN/19	0.01787869
1733/Ck/USA/97	PHC-2020-0545/Ck/CHN/20	0.01791995
MS01/Ck/CHN/19	PHC-2020-0545/Ck/CHN/20	0.01794332
S1133/Ck/CHN/14	526/Ck/CHN/13	0.01794593
T-98/Ck/CHN/06	526/Ck/CHN/13	0.01795934
C78/Ck/CHN/13	SDYT2020/Ck/CHN/20	0.01838068
C78/Ck/CHN/13	AHZJ19/Ck/CHN/19	0.01864506
C78/Ck/CHN/13	PHC-2020-0545/Ck/CHN/20	0.01870970
**R18-37308/Ck/USA/18**	**01224A/Ck/USA/14**	**0.01901625**
S1133/Ck/CHN/14	SDYT2020/Ck/CHN/20	0.01917121
T-98/Ck/CHN/06	SDYT2020/Ck/CHN/20	0.01918462
S1133/Ck/CHN/14	AHZJ19/Ck/CHN/19	0.01943560

T-98/Ck/CHN/06	AHZJ19/Ck/CHN/19	0.01944901
S1133/Ck/CHN/14	PHC-2020-0545/Ck/CHN/20	0.01950023
T-98/Ck/CHN/06	PHC-2020-0545/Ck/CHN/20	0.01951364
526/Ck/CHN/13	SDYT2020/Ck/CHN/20	0.01976695
526/Ck/CHN/13	AHZJ19/Ck/CHN/19	0.02003134
526/Ck/CHN/13	PHC-2020-0545/Ck/CHN/20	0.02009597
**R18-37308/Ck/USA/18**	**R18-38167/Ck/USA/18**	**0.02042379**
1733/Ck/USA/97	LY383/Ck/CHN/16	0.02113511
1733/Ck/USA/97	V-ARV-SD26/Ck/CHN/20	0.02114607
MS01/Ck/CHN/19	LY383/Ck/CHN/16	0.02115848
MS01/Ck/CHN/19	V-ARV-SD26/Ck/CHN/20	0.02116945

The strains displaying the closest evolutionary distance to our isolates, are shown in bold red. The evolutionary distance is shown up to d = 0.02116945.

TableS6. The characteristic amino acid substitutions in the L-class genomic segments.

Strain	Genotypic cluster	Host	Lambda A amino acids											
			31	38	45	57	78	112	116	138	228	300	313	
**S1133**	**I**	**Chicken**	**I**	**D**	**V**	**T**	**A**	**V**	**K**	**S**	**A**	**T**	**K**	
R18-37308*	III	Chicken	**D**	E	**A**	**A**	S	**M**	*	*	T	*	*	
R18-38167	III	Chicken	T	E	*	*	**K**	*	**R**	**A**	T	**N**	**N**	
01224A	III	Chicken	T	E	*	*	S	*	*	*	*	*	*	
1733	I	Chicken	*	*	*	*	*	*	*	*	*	*	*	
C78	I	Chicken	*	*	*	*	*	*	*	*	*	*	*	
MS01	I	Chicken	*	*	*	*	*	*	*	*	*	*	*	
T-98	I	Chicken	*	*	*	*	*	*	*	*	*	*	*	
526	II	Chicken	T	*	*	*	T	*	*	*	*	*	*	
K1600657	II	Chicken	T	*	*	*	T	*	*	*	*	*	*	
SDYT2020	IV	Chicken	A	*	*	*	*	*	*	*	*	*	*	
AHZJ19	IV	Chicken	A	*	*	*	*	*	*	*	*	*	*	
PHC-2020-0545	IV	Chicken	T	*	*	*	*	*	*	*	*	*	*	
LY383	IV	Chicken	T	*	*	*	*	*	*	*	*	P	*	
V-ARV-SD26	IV	Chicken	T	*	*	*	*	*	*	*	*	P	*	
3211-V	V	Chicken	T	K	*	*	*	*	*	*	*	*	*	
22342	VI	Turkey	S	K	*	*	*	*	*	*	*	*	*	
D1007	VII	Partridge	T	K	*	K	V	*	*	*	S	*	*	
091	Outgroup	Duck	T	V	*	*	*	*	*	*	S	*	R	
**Strain**	**Genotypic cluster**	**Host**	**Lambda B amino acids**											
			26	84	88	89	152	233	293	551	638	929	928	1062
**S1133**	**Ia**	**Chicken**	**K**	**Q**	**P**	**D**	**S**	**T**	**V**	**H**	**M**	**V**	**N**	**E**
R18-37308	Id	Chicken	**T**	**R**	D	**E**	T	A	**I**	**Y**	*	S	T	*
K1600657	Id	Chicken	*	*	D	*	T	A	*	*	*	I	T	*
T-98	Ia	Chicken	*	*	*	*	*	*	*	*	*	*	*	*
MS01	Ia	Chicken	*	*	*	*	*	*	*	*	*	*	*	*
1733	Ia	Chicken	*	*	*	*	*	*	*	*	*	*	*	*
C78	Ia	Chicken	*	*	*	*	*	*	*	*	*	*	*	*
LY383	Ib	Chicken	*	*	*	*	*	*	*	*	*	*	*	*
V-ARV-SD26	Ib	Chicken	*	*	*	*	*	*	*	*	*	*	*	*
PHC-2020-0545	Ib	Chicken	*	*	*	*	*	*	*	*	*	*	*	*
SDYT2020	Ib	Chicken	*	*	*	*	*	*	*	*	*	*	*	*
3211-V	Ic	Chicken	*	*	*	*	*	*	*	*	*	A	*	*
R18-38167	II	Chicken	*	*	A	*	A	*	*	*	V	*	*	D
526	II	Chicken	*	*	A	*	A	*	*	*	V	*	*	D
01224A	II	Chicken	*	*	*	*	*	*	*	*	V	*	*	D
22342	III	Turkey	*	*	S	*	*	*	*	*	*	*	*	*
AHZJ19	IV	Chicken	E	*	S	*	*	*	*	*	*	A	*	*
091	V	Duck	D	H	S	*	*	*	*	*	*	A	*	*
**Strain**	**Genotypic cluster**	**Host**	**Lambda C amino acids**											
			23	48	49	50	269	293	472	700	958	1053		

**S1133**	**I**	**Chicken**	**T**	**G**	**N**	**D**	**P**	**N**	**S**	**D**	**I**	**T**
R18-37308	II	Chicken	*	D	R	N	**S**	*	*	*	*	**A**
R18-38167	II	Chicken	**S**	D	R	N	*	**S**	**A**	**N**	**V**	*
K1600657	II	Chicken	*	D	R	N	*	*	*	*	*	*
526	II	Chicken	*	D	R	N	*	*	*	*	*	*
SDYT2020	II	Chicken	*	D	R	N	*	*	*	*	*	*
PHC-2020-0545	II	Chicken	*	D	R	N	*	*	*	*	*	*
01224A	II	Chicken	*	D	R	N	*	*	*	*	*	*
22342	II	Turkey	*	*	Q	*	*	*	*	*	*	*
1733	I	Chicken	*	*	*	*	*	*	*	*	*	*
T-98	I	Chicken	*	*	*	*	*	*	*	*	*	*
MS01	I	Chicken	*	*	*	*	*	*	*	*	*	*
C78	I	Chicken	*	*	*	*	*	*	*	*	*	*
3211-V	I	Chicken	*	N	*	*	*	*	*	E	*	*
LY383	I	Chicken	*	N	*	*	*	*	*	E	*	*
V-ARV-SD26	I	Chicken	*	N	*	*	*	*	*	E	*	*
AHZJ19	I	Chicken	*	H	*	*	*	*	*	E	*	*
091	III	Duck	*	A	Q	*	*	T	*	*	L	*

*Our strains are denoted by red text. Unique amino acid substitutions are referred by bold red text. The highlighted asterisks refer to the amino acids identical to the reference sequence (the vaccine strain: S1133) displayed in bold.

Table S7. Evolutionary divergence estimates between λB sequences of ARV isolates.

Species 1	Species 2	Dist
LY383/Ck/CHN/16	V-ARV-SD26/Ck/CHN/20	0.00000200
1733/Ck/USA/97	MS01/Ck/CHN/19	0.00081200
T-98/Ck/CHN/06	MS01/Ck/CHN/19	0.00243469
1733/Ck/USA/97	T-98/Ck/CHN/06	0.00324469
S1133/Ck/CHN/14	MS01/Ck/CHN/19	0.00325242
1733/Ck/USA/97	S1133/Ck/CHN/14	0.00406242
S1133/Ck/CHN/14	T-98/Ck/CHN/06	0.00568311
MS01/Ck/CHN/19	C78/Ck/CHN/13	0.00570649
1733/Ck/USA/97	C78/Ck/CHN/13	0.00651449
PHC-2020-0545/Ck/CHN/20	SDYT2020/Ck/CHN/20	0.00736260
T-98/Ck/CHN/06	C78/Ck/CHN/13	0.00813919
S1133/Ck/CHN/14	C78/Ck/CHN/13	0.00895691
**R18-37308/Ck/USA/18**	**K1600657/Ck/USA/16**	**0.01081368**
**526/Ck/CHN/13**	**R18-38167/Ck/USA/18**	**0.01221494**
LY383/Ck/CHN/16	SDYT2020/Ck/CHN/20	0.01233924
V-ARV-SD26/Ck/CHN/20	SDYT2020/Ck/CHN/20	0.01233924
LY383/Ck/CHN/16	PHC-2020-0545/Ck/CHN/20	0.01461520
V-ARV-SD26/Ck/CHN/20	PHC-2020-0545/Ck/CHN/20	0.01461520
LY383/Ck/CHN/16	MS01/Ck/CHN/19	0.01634656
V-ARV-SD26/Ck/CHN/20	MS01/Ck/CHN/19	0.01634656
LY383/Ck/CHN/16	1733/Ck/USA/97	0.01715456
V-ARV-SD26/Ck/CHN/20	1733/Ck/USA/97	0.01715456
LY383/Ck/CHN/16	T-98/Ck/CHN/06	0.01877925
V-ARV-SD26/Ck/CHN/20	T-98/Ck/CHN/06	0.01877925
LY383/Ck/CHN/16	S1133/Ck/CHN/14	0.01959698
V-ARV-SD26/Ck/CHN/20	S1133/Ck/CHN/14	0.01959698
SDYT2020/Ck/CHN/20	MS01/Ck/CHN/19	0.02188069
LY383/Ck/CHN/16	C78/Ck/CHN/13	0.02204705
V-ARV-SD26/Ck/CHN/20	C78/Ck/CHN/13	0.02204705
SDYT2020/Ck/CHN/20	1733/Ck/USA/97	0.02268869
MS01/Ck/CHN/19	K1600657/Ck/USA/16	0.02333990
1733/Ck/USA/97	K1600657/Ck/USA/16	0.02414790
PHC-2020-0545/Ck/CHN/20	MS01/Ck/CHN/19	0.02415665
SDYT2020/Ck/CHN/20	T-98/Ck/CHN/06	0.02431338
PHC-2020-0545/Ck/CHN/20	1733/Ck/USA/97	0.02496465
SDYT2020/Ck/CHN/20	S1133/Ck/CHN/14	0.02513110

The strains displaying the closest evolutionary distance to our isolates, are shown in bold red. The evolutionary distance is shown up to *d* = 0.02513110.

Table S8. Evolutionary divergence estimates between λC sequences of ARV isolates.

Species 1	Species 2	Dist
T-98/Ck/CHN/06	MS01/Ck/CHN/19	0.00081872
1733/Ck/USA/97	MS01/Ck/CHN/19	0.00081894
1733/Ck/USA/97	T-98/Ck/CHN/06	0.00157096
S1133/Ck/CHN/14	MS01/Ck/CHN/19	0.00159453
LY383/Ck/CHN/16	V-ARV-SD26/Ck/CHN/20	0.00236270
T-98/Ck/CHN/06	S1133/Ck/CHN/14	0.00236878
1733/Ck/USA/97	S1133/Ck/CHN/14	0.00236900
MS01/Ck/CHN/19	C78/Ck/CHN/13	0.00634325
T-98/Ck/CHN/06	C78/Ck/CHN/13	0.00713973
1733/Ck/USA/97	C78/Ck/CHN/13	0.00713996
S1133/Ck/CHN/14	C78/Ck/CHN/13	0.00791554
V-ARV-SD26/Ck/CHN/20	AHZJ19/Ck/CHN/19	0.01508701
LY383/Ck/CHN/16	AHZJ19/Ck/CHN/19	0.01587869
3211-V/Ck/HUN/02	AHZJ19/Ck/CHN/19	0.02550047
3211-V/Ck/HUN/02	V-ARV-SD26/Ck/CHN/20	0.02626073
3211-V/Ck/HUN/02	LY383/Ck/CHN/16	0.02705241
**R18-37308/Ck/USA/18**	**R18-38167/Ck/USA/18**	**0.02743100**
01224A/Ck/USA/14	PHC-2020-0545/Ck/CHN/20	0.02846770
R18-38167/Ck/USA/18	K1600657/Ck/USA/16	0.03387652
R18-37308/Ck/USA/18	K1600657/Ck/USA/16	0.03414080
526/Ck/CHN/13	K1600657/Ck/USA/16	0.03458699
526/Ck/CHN/13	SDYT2020/Ck/CHN/20	0.03541316
R18-38167/Ck/USA/18	526/Ck/CHN/13	0.03755216
R18-37308/Ck/USA/18	526/Ck/CHN/13	0.03781644
K1600657/Ck/USA/16	SDYT2020/Ck/CHN/20	0.03861343
PHC-2020-0545/Ck/CHN/20	SDYT2020/Ck/CHN/20	0.03948322
526/Ck/CHN/13	PHC-2020-0545/Ck/CHN/20	0.04019064
R18-38167/Ck/USA/18	SDYT2020/Ck/CHN/20	0.04157860
R18-37308/Ck/USA/18	SDYT2020/Ck/CHN/20	0.04184288
K1600657/Ck/USA/16	PHC-2020-0545/Ck/CHN/20	0.04339091
R18-38167/Ck/USA/18	PHC-2020-0545/Ck/CHN/20	0.04635608
R18-37308/Ck/USA/18	PHC-2020-0545/Ck/CHN/20	0.04662036
01224A/Ck/USA/14	SDYT2020/Ck/CHN/20	0.04697181
526/Ck/CHN/13	01224A/Ck/USA/14	0.04767923
K1600657/Ck/USA/16	01224A/Ck/USA/14	0.05087950

The strains displaying the closest evolutionary distance to our isolates, are shown in bold red.
The evolutionary distance is shown up to d = 0.05087950.

Table S9. Evolutionary divergence estimates between μA sequences of ARV isolates.

Species 1	Species 2	Dist
V-ARV-SD26/Ck/CHN/20	LY383/Ck/CHN/16	0.00000200
1733/Ck/USA/97	C78/Ck/CHN/13	0.00271224
C78/Ck/CHN/13	MS01/Ck/CHN/19	0.00271437
1733/Ck/USA/97	MS01/Ck/CHN/19	0.00271587
S1133/Ck/CHN/14	1733/Ck/USA/97	0.00407552
S1133/Ck/CHN/14	C78/Ck/CHN/13	0.00407603
S1133/Ck/CHN/14	MS01/Ck/CHN/19	0.00407965
T-98/Ck/CHN/06	MS01/Ck/CHN/19	0.00544851
C78/Ck/CHN/13	T-98/Ck/CHN/06	0.00816088
1733/Ck/USA/97	T-98/Ck/CHN/06	0.00816238
AHZJ19/Ck/CHN/19	PHC-2020-0545/Ck/CHN/20	0.00947951
S1133/Ck/CHN/14	T-98/Ck/CHN/06	0.00952616
1733/Ck/USA/97	SDYT2020/Ck/CHN/20	0.00955087
C78/Ck/CHN/13	SDYT2020/Ck/CHN/20	0.00955137
MS01/Ck/CHN/19	SDYT2020/Ck/CHN/20	0.00955499
S1133/Ck/CHN/14	SDYT2020/Ck/CHN/20	0.01091265
T-98/Ck/CHN/06	SDYT2020/Ck/CHN/20	0.01500150
**R18-37308/Ck/USA/18**	**01224A/Ck/USA/14**	**0.02196731**
**R18-38167/Ck/USA/18**	**01224A/Ck/USA/14**	**0.02350353**
**R18-38167/Ck/USA/18**	**R18-37308/Ck/USA/18**	**0.02880049**
V-ARV-SD26/Ck/CHN/20	1733/Ck/USA/97	0.03024178
LY383/Ck/CHN/16	1733/Ck/USA/97	0.03024178
V-ARV-SD26/Ck/CHN/20	C78/Ck/CHN/13	0.03024228
LY383/Ck/CHN/16	C78/Ck/CHN/13	0.03024228
V-ARV-SD26/Ck/CHN/20	MS01/Ck/CHN/19	0.03024590
LY383/Ck/CHN/16	MS01/Ck/CHN/19	0.03024590
V-ARV-SD26/Ck/CHN/20	SDYT2020/Ck/CHN/20	0.03046452
LY383/Ck/CHN/16	SDYT2020/Ck/CHN/20	0.03046452
V-ARV-SD26/Ck/CHN/20	S1133/Ck/CHN/14	0.03160356
LY383/Ck/CHN/16	S1133/Ck/CHN/14	0.03160356
526/Ck/CHN/13	1733/Ck/USA/97	0.03175511
526/Ck/CHN/13	C78/Ck/CHN/13	0.03175561
526/Ck/CHN/13	MS01/Ck/CHN/19	0.03175923
526/Ck/CHN/13	SDYT2020/Ck/CHN/20	0.03197785
V-ARV-SD26/Ck/CHN/20	R18-38167/Ck/USA/18	0.03225816
LY383/Ck/CHN/16	R18-38167/Ck/USA/18	0.03225816
V-ARV-SD26/Ck/CHN/20	01224A/Ck/USA/14	0.03310999
LY383/Ck/CHN/16	01224A/Ck/USA/14	0.03310999

The strains displaying the closest evolutionary distance to our isolates, are shown in bold red. The evolutionary distance is shown up to d = 0.03310999.

Table S10. The characteristic amino acid substitutions in the M-class genomic segments.

Strain	Genotypic cluster	Host	μA amino acids							
			44	55	170	280	377	393	417	598
**S1133**	**I**	**Chicken**	**T**	**T**	**A**	**P**	**Y**	**A**	**K**	**S**
R18-37308	IIIa	Chicken	*	**I**	V	*	H	**T**	**R**	**T**
R18-38167	IIIa	Chicken	**P**	*	**I**	**L**	H	*	*	**G**
01224A	IIIa	Chicken	*	*	V	*	H	*	*	*
LY383	IIIa	Chicken	*	*	V	*	*	*	*	N
V-ARV-SD26	IIIa	Chicken	*	*	V	*	*	*	*	N
MS01	I	Chicken	*	*	V	*	*	*	*	*
T-98	I	Chicken	*	*	V	*	*	*	*	*
C78	I	Chicken	*	*	V	*	*	*	*	*
1733	I	Chicken	*	*	V	*	*	*	*	*
SDYT2020	I	Chicken	*	*	*	*	*	*	*	*
K1600657	IIa	Chicken	*	*	V	*	*	*	*	R
526	IIb	Chicken	*	*	V	*	*	*	*	*
3211-V	IIc	Chicken	*	*	V	*	*	*	*	N
D1007	IIc	Partridge	*	*	V	*	*	*	*	N
PHC-2020-0545	IIc	Chicken	*	*	V	*	*	*	*	N
AHZJ19	IIc	Chicken	*	*	V	*	*	*	*	N
22342	IIIb	Turkey	*	*	V	*	*	*	*	*
091	Outgroup	Duck	*	*	V	*	*	S	*	P

Strain	Genotypic cluster	Host	μB amino acids								
			80	109	139	160	234	272	460	484	593
**S1133**	**Ia**	**Chicken**	**G**	**S**	**N**	**R**	**E**	**A**	**S**	**V**	**I**
R18-37308	II	Chicken	S	*	S	K	D	T	**L**	**T**	**V**
526	II	Chicken	S	*	S	K	D	T	*	*	*
R18-38167	II	Chicken	S	**T**	S	K	D	T	*	*	*
K1600657	II	Chicken	S	A	S	K	D	T	*	*	*
01224A	II	Chicken	S	*	S	K	D	T	*	*	*
LY383	II	Chicken	S	*	S	K	D	T	*	A	*
V-ARV-SD26	II	Chicken	S	*	S	K	D	T	*	A	*
T-98	I	Chicken	*	*	*	*	*	*	*	*	*
C78	I	Chicken	*	*	*	*	*	*	*	*	*
MS01	I	Chicken	*	*	*	*	*	*	*	*	*
1733	I	Chicken	*	*	*	*	*	*	*	*	*
22342	I	Turkey	*	*	*	*	*	*	*	*	*
PHC-2020-0545	III	Chicken	*	A	*	Q	*	*	A	*	*
SDYT2020	III	Chicken	*	A	*	Q	*	*	A	*	F
AHZJ19	IV	Chicken	A	*	*	Q	*	*	*	A	F
3211-V	V	Chicken	*	*	*	Q	*	*	A	A	F
091	VI	Duck	*	*	*	Q	*	*	N	W	L
D1007	VI	Partridge	*	*	*	Q	*	*	N	W	L

Strain	Host	μNS amino acids

	Genotypic cluster		253	300	590	618
**S1133**	**I**	**Chicken**	**S**	**A**	**V**	**S**
R18-37308	II	Chicken	A	**S**	M	L
R18-38167	II	Chicken	**T**	*	M	L
LY383	II	Chicken	A	*	*	P
V-ARV-SD26	II	Chicken	A	*	*	P
3211-V	II	Chicken	A	*	*	P
AHZJ19	II	Chicken	A	*	*	P
SDYT2020	II	Chicken	A	*	*	*
D1007	II	Partridge	A	*	*	*
PHC-2020-0545	II	Chicken	A	*	*	P
1733	I	Chicken	*	*	*	*
C78	I	Chicken	*	*	*	*
T-98	I	Chicken	*	*	*	*
MS01	I	Chicken	*	*	*	*
K1600657	I	Chicken	*	*	A	*
526	I	Chicken	*	*	A	*
01224A	I	Chicken	*	*	*	*
22342	I	Turkey	*	*	*	*
091	III	Duck	P	*	*	*

*Our strains are denoted by red text. Unique amino acid substitutions are referred by bold red text. The highlighted asterisks refer to the amino acids identical to the reference sequence (the vaccine strain: S1133) displayed in bold.

Table S11. Evolutionary divergence estimates between μB sequences of ARV isolates.

Species 1	Species 2	Dist
V-ARV-SD26/Ck/CHN/20	LY383/Ck/CHN/16	0.00138540
MS01/Ck/CHN/19	1733/Ck/USA/97	0.00277034
MS01/Ck/CHN/19	S1133/Ck/CHN/14	0.00415563
T-98/Ck/CHN/06	MS01/Ck/CHN/19	0.00571019
1733/Ck/USA/97	S1133/Ck/CHN/14	0.00692197
C78/Ck/CHN/13	MS01/Ck/CHN/19	0.00693800
T-98/Ck/CHN/06	1733/Ck/USA/97	0.00847853
C78/Ck/CHN/13	1733/Ck/USA/97	0.00970634
PHC-2020-0545/Ck/CHN/20	SDYT2020/Ck/CHN/20	0.00971786
T-98/Ck/CHN/06	S1133/Ck/CHN/14	0.00986382
**R18-38167/Ck/USA/18**	**K1600657/Ck/USA/16**	**0.01044857**
C78/Ck/CHN/13	S1133/Ck/CHN/14	0.01109163
T-98/Ck/CHN/06	C78/Ck/CHN/13	0.01264418
01224A/Ck/USA/14	K1600657/Ck/USA/16	0.01423907
01224A/Ck/USA/14	R18-38167/Ck/USA/18	0.01611146
**V-ARV-SD26/Ck/CHN/20**	**R18-37308/Ck/USA/18**	**0.01628661**
LY383/Ck/CHN/16	R18-37308/Ck/USA/18	0.01767001
V-ARV-SD26/Ck/CHN/20	526/Ck/CHN/13	0.01973738
LY383/Ck/CHN/16	526/Ck/CHN/13	0.02112078
R18-37308/Ck/USA/18	526/Ck/CHN/13	0.02120220
K1600657/Ck/USA/16	V-ARV-SD26/Ck/CHN/20	0.02407793
01224A/Ck/USA/14	V-ARV-SD26/Ck/CHN/20	0.02540462
K1600657/Ck/USA/16	LY383/Ck/CHN/16	0.02546133
R18-38167/Ck/USA/18	V-ARV-SD26/Ck/CHN/20	0.02595032
01224A/Ck/USA/14	LY383/Ck/CHN/16	0.02678802
R18-38167/Ck/USA/18	LY383/Ck/CHN/16	0.02733372
K1600657/Ck/USA/16	R18-37308/Ck/USA/18	0.03119979
01224A/Ck/USA/14	R18-37308/Ck/USA/18	0.03252648
R18-38167/Ck/USA/18	R18-37308/Ck/USA/18	0.03307218
MS01/Ck/CHN/19	22342/Tk/USA/13	0.03395056
K1600657/Ck/USA/16	526/Ck/CHN/13	0.03465056
01224A/Ck/USA/14	526/Ck/CHN/13	0.03597725
T-98/Ck/CHN/06	22342/Tk/USA/13	0.03631329
R18-38167/Ck/USA/18	526/Ck/CHN/13	0.03652295
1733/Ck/USA/97	22342/Tk/USA/13	0.03671890
S1133/Ck/CHN/14	22342/Tk/USA/13	0.03810419
C78/Ck/CHN/13	22342/Tk/USA/13	0.04088456

The strains displaying the closest evolutionary distance to our isolates, are shown in bold red. The evolutionary distance is shown up to d = 0.04088456.

Table S12. Evolutionary divergence estimates between μNS sequences of ARV isolates.

Species 1	Species 2	Dist
T-98/Ck/CHN/06	MS01/Ck/CHN/19	0.00000200
S1133/Ck/CHN/14	T-98/Ck/CHN/06	0.00157663
S1133/Ck/CHN/14	MS01/Ck/CHN/19	0.00157663
1733/Ck/USA/97	S1133/Ck/CHN/14	0.00315190
V-ARV-SD26/Ck/CHN/20	LY383/Ck/CHN/16	0.00320242
1733/Ck/USA/97	T-98/Ck/CHN/06	0.00472653
1733/Ck/USA/97	MS01/Ck/CHN/19	0.00472653
S1133/Ck/CHN/14	C78/Ck/CHN/13	0.00472737
T-98/Ck/CHN/06	C78/Ck/CHN/13	0.00630000
MS01/Ck/CHN/19	C78/Ck/CHN/13	0.00630000
1733/Ck/USA/97	C78/Ck/CHN/13	0.00787727
**R18-37308/Ck/USA/18**	**R18-38167/Ck/USA/18**	**0.01117783**
R18-38167/Ck/USA/18	V-ARV-SD26/Ck/CHN/20	0.01938768
R18-37308/Ck/USA/18	V-ARV-SD26/Ck/CHN/20	0.02105670
R18-38167/Ck/USA/18	LY383/Ck/CHN/16	0.02258809
R18-37308/Ck/USA/18	LY383/Ck/CHN/16	0.02425712
V-ARV-SD26/Ck/CHN/20	3211-V/Ck/HUN/02	0.02456582
SDYT2020/Ck/CHN/20	AHZJ19/Ck/CHN/19	0.02606495
LY383/Ck/CHN/16	3211-V/Ck/HUN/02	0.02776624
AHZJ19/Ck/CHN/19	V-ARV-SD26/Ck/CHN/20	0.03392053
R18-38167/Ck/USA/18	3211-V/Ck/HUN/02	0.03442540
K1600657/Ck/USA/16	526/Ck/CHN/13	0.03541044
R18-37308/Ck/USA/18	3211-V/Ck/HUN/02	0.03609442
SDYT2020/Ck/CHN/20	V-ARV-SD26/Ck/CHN/20	0.03618584
AHZJ19/Ck/CHN/19	LY383/Ck/CHN/16	0.03712095
SDYT2020/Ck/CHN/20	LY383/Ck/CHN/16	0.03938626
AHZJ19/Ck/CHN/19	3211-V/Ck/HUN/02	0.04291961
AHZJ19/Ck/CHN/19	R18-38167/Ck/USA/18	0.04378011
S1133/Ck/CHN/14	526/Ck/CHN/13	0.04451880
SDYT2020/Ck/CHN/20	3211-V/Ck/HUN/02	0.04518493
AHZJ19/Ck/CHN/19	R18-37308/Ck/USA/18	0.04544914
SDYT2020/Ck/CHN/20	R18-38167/Ck/USA/18	0.04604543
T-98/Ck/CHN/06	526/Ck/CHN/13	0.04609343
MS01/Ck/CHN/19	526/Ck/CHN/13	0.04609343
1733/Ck/USA/97	526/Ck/CHN/13	0.04766670
SDYT2020/Ck/CHN/20	R18-37308/Ck/USA/18	0.04771445
C78/Ck/CHN/13	526/Ck/CHN/13	0.04924418
PHC-2020-0545/Ck/CHN/20	3211-V/Ck/HUN/02	0.05001362

The strains displaying the closest evolutionary distance to our isolates, are shown in bold red. The evolutionary distance is shown up to *d* = 0.05001362.

Table S13. Evolutionary divergence estimates between P10 sequences of ARV isolates.

Species 1	Species 2	Dist
LY383/Ck/CHN/16	V-ARV-SD26/Ck/CHN/20	0.00000000
1733/Ck/USA/97	S1133/Ck/CHN/14	0.00000000
C78/Ck/CHN/13	1733/Ck/USA/97	0.00000000
C78/Ck/CHN/13	S1133/Ck/CHN/14	0.00000000
MS01/Ck/CHN/19	C78/Ck/CHN/13	0.00000000
MS01/Ck/CHN/19	1733/Ck/USA/97	0.00000000
MS01/Ck/CHN/19	S1133/Ck/CHN/14	0.00000000
T-98/Ck/CHN/06	MS01/Ck/CHN/19	0.00000000
T-98/Ck/CHN/06	C78/Ck/CHN/13	0.00000000
T-98/Ck/CHN/06	1733/Ck/USA/97	0.00000000
T-98/Ck/CHN/06	S1133/Ck/CHN/14	0.00000000
**R18-38167/Ck/USA/18**	**LY383/Ck/CHN/16**	**0.01389353**
**R18-38167/Ck/USA/18**	**V-ARV-SD26/Ck/CHN/20**	**0.01389353**
01224A/Ck/USA/14	AHZJ19/Ck/CHN/19	0.02981907
T-98/Ck/CHN/06	526/Ck/CHN/13	0.04157661
MS01/Ck/CHN/19	526/Ck/CHN/13	0.04157661
C78/Ck/CHN/13	526/Ck/CHN/13	0.04157661
1733/Ck/USA/97	526/Ck/CHN/13	0.04157661
S1133/Ck/CHN/14	526/Ck/CHN/13	0.04157661
**R18-37308/Ck/USA/18**	**R18-38167/Ck/USA/18**	**0.06560132**
R18-37308/Ck/USA/18	LY383/Ck/CHN/16	0.07131342
R18-37308/Ck/USA/18	V-ARV-SD26/Ck/CHN/20	0.07131342
SDYT2020/Ck/CHN/20	K1600657/Ck/USA/16	0.08304182
SDYT2020/Ck/CHN/20	3211-V/Ck/HUN/02	0.14605930
22342/Tk/USA/13	D1007/PRT/HUN/08	0.17585934
LY383/Ck/CHN/16	3211-V/Ck/HUN/02	0.17649349
V-ARV-SD26/Ck/CHN/20	3211-V/Ck/HUN/02	0.17649349
LY383/Ck/CHN/16	SDYT2020/Ck/CHN/20	0.17733352
V-ARV-SD26/Ck/CHN/20	SDYT2020/Ck/CHN/20	0.17733352
3211-V/Ck/HUN/02	K1600657/Ck/USA/16	0.18983750
R18-38167/Ck/USA/18	3211-V/Ck/HUN/02	0.19011943
R18-38167/Ck/USA/18	SDYT2020/Ck/CHN/20	0.19095946
PHC-2020-0545/Ck/CHN/20	D1007/PRT/HUN/08	0.19893470
LY383/Ck/CHN/16	01224A/Ck/USA/14	0.21113664
V-ARV-SD26/Ck/CHN/20	01224A/Ck/USA/14	0.21113664
LY383/Ck/CHN/16	K1600657/Ck/USA/16	0.22111173
V-ARV-SD26/Ck/CHN/20	K1600657/Ck/USA/16	0.22111173
R18-38167/Ck/USA/18	01224A/Ck/USA/14	0.22476258
LY383/Ck/CHN/16	AHZJ19/Ck/CHN/19	0.22673994

The strains displaying the closest evolutionary distance to our isolates, are shown in bold red. The evolutionary distance is shown up to d = 0.22673994.

Table 14. The characteristic amino acid substitutions in the S-class genomic segments.

Strain	Genotypic cluster			Host	S1 ORFs																	
					P10			P17			σC											
	P10	P17	σC		67	77	93	34	96	99	33	54	101	108	119	120	121	123	133	144	150	256
**S1133**	**I**	**I**	**I**	**Chicken**	**A**	**V**	**M**	**G**	**I**	**L**	**I**	**I**	**T**	**S**	**T**	**T**	**V**	**G**	**S**	**D**	**K**	**T**
R18-37308	V	V	VI	Chicken	**V**	**A**	A	**N**	**V**	**D**	*	**T**	**V**	R	E	**K**	*	A	**A**	**G**	Q	*
R18-38167	V	V	VI	Chicken	*	*	A	D	*	N	V	A	A	**H**	**K**	E	A	**V**	T	S	**E**	V
LY383	V	V	VI	Chicken	*	*	A	D	*	N	*	A	A	R	R	E	*	A	T	S	Q	I
V-ARV-SD26	V	V	VI	Chicken	*	*	A	D	*	N	*	A	A	R	R	E	*	A	T	S	Q	I
1733	I	I	I	Chicken	*	*	*	*	*	*	*	*	*	*	*	*	*	*	*	*	*	*
C78	I	I	I	Chicken	*	*	*	*	*	*	*	*	*	*	*	*	*	*	*	*	*	*
MS01	I	I	I	Chicken	*	*	*	*	*	*	*	*	*	*	*	*	*	*	*	*	*	*
T-98	I	I	I	Chicken	*	*	*	*	*	*	*	*	*	*	*	*	*	*	*	*	*	*
526	I	I	I	Chicken	*	*	*	*	*	*	*	V	*	*	*	A	*	R	*	*	*	*
Pycno-1	IIa	-	-	Bulbul	*	*	R	-	-	-	-	-	-	-	-	-	-	-	-	-	-	-
D1007	IIb	II	II	Partridge	*	*	V	A	*	*	L	L	S	T	*	S	*	N	*	*	*	*
22342	IIb	II	II	Turkey	*	*	V	A	*	*	*	L	L	T	*	S	*	N	*	*	*	*
PHC-2020-0545	IIb	II	II	Chicken	*	*	I	A	*	*	*	L	S	A	P	A	*	A	G	*	*	*
AHZJ19	III	III	III	Chicken	*	I	R	D	*	*	*	L	S	*	S	S	*	T	R	*	A	*
01224A	III	III	III	Chicken	*	I	R	D	*	*	*	L	S	A	S	S	*	N	R	*	A	*
3211-V	IV	IV	IV	Chicken	*	I	E	D	*	S	L	V	*	D	Q	S	*	T	*	S	T	I
K1600657	IV	-	V	Chicken	*	*	G	-	-	-	L	V	S	N	S	M	*	*	R	S	S	I
SDYT2020	IV	IV	V	Chicken	*	*	G	*	*	V	L	A	S	N	S	H	L	*	R	S	T	I

Strain	Genotypic cluster	Host	σA amino acids				
			119	225	229	315	365
**S1133**	**I**	**Chicken**	**S**	**V**	**T**	**P**	**L**

R18-37308	I	Chicken	P	*	N	*	I
R18-38167	I	Chicken	*	I	*	S	*
PHC-2020-0545	I	Chicken	*	*	*	*	*
AHZJ19	I	Chicken	*	*	*	*	*
01224A	I	Chicken	*	*	*	*	F
LY383	I	Chicken	*	*	*	*	*
V-ARV-SD26	I	Chicken	*	*	*	*	*
526	I	Chicken	*	*	*	*	*
C78	I	Chicken	*	*	*	*	*
T-98	I	Chicken	*	*	*	*	*
MS01	I	Chicken	*	*	*	*	*
1733	I	Chicken	*	*	*	*	*
SDYT2020	II	Chicken	*	*	*	*	*
3211-V	II	Chicken	*	*	*	*	*
D1007	II	Partridge	*	*	*	*	*
22342	III	Turkey	*	*	*	*	*
091	IV	Duck	*	*	S	*	*

**Strain**	**Genotypic cluster**	**Host**	**σB amino acids**												
			21	34	127	134	144	193	194	198	229	277	344	348	350
**S1133**	**Ia**	**Chicken**	**K**	**T**	**N**	**D**	**S**	**S**	**L**	**S**	**S**	**H**	**A**	**G**	**V**
R18-37308	Ic	Chicken	*	**A**	*	*	**G**	*	*	*	*	N	*	**D**	**T**
R18-38167	Ib	Chicken	**Q**	*	**K**	**G**	*	**A**	**V**	**A**	A	Q	**P**	*	*
K1600657	Ib	Chicken	*	*	*	*	*	*	*	*	A	Q	*	*	*
LY383	Ib	Chicken	*	*	*	*	*	*	*	*	A	Q	*	*	*

V-ARV-SD26	Ib	Chicken	*	*	*	*	*	*	*	*	A	Q	*	*	*
SDYT2020	Ib	Chicken	*	*	*	*	*	*	*	*	A	Q	*	*	*
AHZJ19	Ib	Chicken	*	*	*	*	*	*	*	*	A	Q	*	*	*
PHC-2020-0545	Ib	Chicken	*	*	*	*	*	*	*	*	A	Q	*	*	*
T-98	Ia	Chicken	*	*	*	*	*	*	*	*	*	*	*	*	*
1733	Ia	Chicken	*	*	*	*	*	*	*	*	*	*	*	*	*
MS01	Ia	Chicken	*	*	*	*	*	*	*	*	*	*	*	*	*
C78	Ia	Chicken	*	*	*	*	*	*	*	*	*	*	*	*	*
3211-V	Ia	Chicken	*	*	*	*	*	*	*	*	*	N	*	*	*
01224A	Ia	Chicken	*	*	*	*	*	*	*	*	*	*	T	*	*
526	Ia	Chicken	*	*	*	*	*	*	*	*	*	N	T	*	A
D1007	II	Partridge	*	*	*	*	*	*	*	*	P	*	*	*	M
22342	III	Turkey	R	V	E	*	*	*	*	H	P	*	E	R	A
091	III	Duck	R	I	T	*	*	M	*	H	P	N	T	*	S

Strain	Genotypic cluster	Host	σNS amino acids						
			25	82	120	152	257	273	350
**S1133**	**I**	**Chicken**	**F**	**L**	**V**	**R**	**M**	**T**	**M**
R18-37308	II	Chicken	Y	*	L	H	F	V	T
R18-38167	II	Chicken	Y	**M**	**I**	H	F	V	T
526	II	Chicken	*	*	L	H	F	V	T
01224A	II	Chicken	*	*	L	H	F	V	T
LY383	II	Chicken	*	*	L	H	F	V	T
V-ARV-SD26	II	Chicken	*	*	L	H	F	V	T
SDYT2020	II	Chicken	*	*	L	H	F	V	T

K1600657	II	Chicken	*	*	L	H	F	V	T
T-98	I	Chicken	*	*	*	*	*	*	*
C78	I	Chicken	*	*	*	*	*	*	*
1733	I	Chicken	*	*	*	*	*	*	*
MS01	I	Chicken	*	*	*	*	*	*	*
AHZJ19	III	Chicken	*	*	A	Q	*	*	*
3211-V	III	Chicken	*	*	*	*	*	*	L
PHC-2020-0545	III	Chicken	*	*	*	*	*	*	*
D1007	IV	Partridge	*	*	H	*	*	N	*
22342	IV	Turkey	*	*	Y	*	*	S	*
091	V	Duck	*	*	*	Q	*	*	*

*Our strains are denoted by red text. Unique amino acid substitutions are referred by bold red text. The highlighted asterisks refer to the amino acids identical to the reference sequence (the vaccine strain: S1133) displayed in bold.

Table S15. Evolutionary divergence estimates between P17 sequences of ARV isolates.

Species 1	Species 2	Dist
V-ARV-SD26/Ck/CHN/20	LY383/Ck/CHN/16	0.00000200
T-98/Ck/CHN/06	1733/Ck/USA/97	0.00000200
T-98/Ck/CHN/06	MS01/Ck/CHN/19	0.00000300
1733/Ck/USA/97	MS01/Ck/CHN/19	0.00000300
S1133/Ck/CHN/14	MS01/Ck/CHN/19	0.00677441
S1133/Ck/CHN/14	T-98/Ck/CHN/06	0.00677541
S1133/Ck/CHN/14	1733/Ck/USA/97	0.00677541
C78/Ck/CHN/13	MS01/Ck/CHN/19	0.00677714
C78/Ck/CHN/13	T-98/Ck/CHN/06	0.00677814
C78/Ck/CHN/13	1733/Ck/USA/97	0.00677814
C78/Ck/CHN/13	S1133/Ck/CHN/14	0.01354755
**V-ARV-SD26/Ck/CHN/20**	**R18-38167/Ck/USA/18**	**0.03802317**
**LY383/Ck/CHN/16**	**R18-38167/Ck/USA/18**	**0.03802317**
**R18-37308/Ck/USA/18**	**R18-38167/Ck/USA/18**	**0.05695521**
R18-37308/Ck/USA/18	V-ARV-SD26/Ck/CHN/20	0.06068615
R18-37308/Ck/USA/18	LY383/Ck/CHN/16	0.06068615
D1007/PRT/HUN/08	22342/Tk/USA/13	0.11118072
01224A/Ck/USA/14	AHZJ19/Ck/CHN/19	0.11404780
526/Ck/CHN/13	C78/Ck/CHN/13	0.14736841
526/Ck/CHN/13	MS01/Ck/CHN/19	0.15414355
526/Ck/CHN/13	T-98/Ck/CHN/06	0.15414455
526/Ck/CHN/13	1733/Ck/USA/97	0.15414455
526/Ck/CHN/13	S1133/Ck/CHN/14	0.16091396
SDYT2020/Ck/CHN/20	3211-V/Ck/HUN/02	0.27657625
PHC-2020-0545/Ck/CHN/20	22342/Tk/USA/13	0.29041791
PHC-2020-0545/Ck/CHN/20	D1007/PRT/HUN/08	0.32455917
R18-37308/Ck/USA/18	3211-V/Ck/HUN/02	0.40778189
R18-37308/Ck/USA/18	SDYT2020/Ck/CHN/20	0.40920637
R18-38167/Ck/USA/18	3211-V/Ck/HUN/02	0.42163938
R18-38167/Ck/USA/18	SDYT2020/Ck/CHN/20	0.42306386
V-ARV-SD26/Ck/CHN/20	3211-V/Ck/HUN/02	0.42537031
LY383/Ck/CHN/16	3211-V/Ck/HUN/02	0.42537031
V-ARV-SD26/Ck/CHN/20	SDYT2020/Ck/CHN/20	0.42679479
LY383/Ck/CHN/16	SDYT2020/Ck/CHN/20	0.42679479
3211-V/Ck/HUN/02	AHZJ19/Ck/CHN/19	0.54586437
SDYT2020/Ck/CHN/20	AHZJ19/Ck/CHN/19	0.54728885
PHC-2020-0545/Ck/CHN/20	AHZJ19/Ck/CHN/19	0.55600156
R18-37308/Ck/USA/18	AHZJ19/Ck/CHN/19	0.55866226
R18-38167/Ck/USA/18	AHZJ19/Ck/CHN/19	0.57251975

The strains displaying the closest evolutionary distance to our isolates, are shown in bold red. The evolutionary distance is shown up to d = 0.57251975.

Table S16. Evolutionary divergence estimates between σA sequences of ARV isolates.

Species 1	Species 2	Dist
V-ARV-SD26/Ck/CHN/20	LY383/Ck/CHN/16	0.00000200
T-98/Ck/CHN/06	S1133/Ck/CHN/14	0.00000200
MS01/Ck/CHN/19	T-98/Ck/CHN/06	0.00236748
MS01/Ck/CHN/19	S1133/Ck/CHN/14	0.00236748
1733/Ck/USA/97	T-98/Ck/CHN/06	0.00237387
1733/Ck/USA/97	S1133/Ck/CHN/14	0.00237387
PHC-2020-0545/Ck/CHN/20	AHZJ19/Ck/CHN/19	0.00237598
MS01/Ck/CHN/19	1733/Ck/USA/97	0.00473534
T-98/Ck/CHN/06	C78/Ck/CHN/13	0.00714298
S1133/Ck/CHN/14	C78/Ck/CHN/13	0.00714298
**V-ARV-SD26/Ck/CHN/20**	**R18-38167/Ck/USA/18**	**0.00947308**
**LY383/Ck/CHN/16**	**R18-38167/Ck/USA/18**	**0.00947308**
MS01/Ck/CHN/19	C78/Ck/CHN/13	0.00950645
1733/Ck/USA/97	C78/Ck/CHN/13	0.00951285
3211-V/Ck/HUN/02	SDYT2020/Ck/CHN/20	0.01207259
AHZJ19/Ck/CHN/19	R18-38167/Ck/USA/18	0.01425852
**R18-37308/Ck/USA/18**	**R18-38167/Ck/USA/18**	**0.01425894**
AHZJ19/Ck/CHN/19	V-ARV-SD26/Ck/CHN/20	0.01426347
AHZJ19/Ck/CHN/19	LY383/Ck/CHN/16	0.01426347
R18-37308/Ck/USA/18	V-ARV-SD26/Ck/CHN/20	0.01426389
R18-37308/Ck/USA/18	LY383/Ck/CHN/16	0.01426389
T-98/Ck/CHN/06	R18-38167/Ck/USA/18	0.01427675
S1133/Ck/CHN/14	R18-38167/Ck/USA/18	0.01427675
V-ARV-SD26/Ck/CHN/20	T-98/Ck/CHN/06	0.01428370
LY383/Ck/CHN/16	T-98/Ck/CHN/06	0.01428370
V-ARV-SD26/Ck/CHN/20	S1133/Ck/CHN/14	0.01428370
LY383/Ck/CHN/16	S1133/Ck/CHN/14	0.01428370
R18-37308/Ck/USA/18	AHZJ19/Ck/CHN/19	0.01431613
PHC-2020-0545/Ck/CHN/20	R18-38167/Ck/USA/18	0.01663250
PHC-2020-0545/Ck/CHN/20	V-ARV-SD26/Ck/CHN/20	0.01663745
PHC-2020-0545/Ck/CHN/20	LY383/Ck/CHN/16	0.01663745
MS01/Ck/CHN/19	R18-38167/Ck/USA/18	0.01664023
1733/Ck/USA/97	R18-38167/Ck/USA/18	0.01664662
V-ARV-SD26/Ck/CHN/20	MS01/Ck/CHN/19	0.01664718
LY383/Ck/CHN/16	MS01/Ck/CHN/19	0.01664718
V-ARV-SD26/Ck/CHN/20	1733/Ck/USA/97	0.01665357
LY383/Ck/CHN/16	1733/Ck/USA/97	0.01665357
R18-37308/Ck/USA/18	PHC-2020-0545/Ck/CHN/20	0.01669011
AHZJ19/Ck/CHN/19	T-98/Ck/CHN/06	0.01906914

The strains displaying the closest evolutionary distance to our isolates, are shown in bold red. The evolutionary distance is shown up to d = 0.01906914.

Table S17. Evolutionary divergence estimates between σB sequences of ARV isolates.

Species 1	Species 2	Dist
LY383/Ck/CHN/16	V-ARV-SD26/Ck/CHN/20	0.00007188
T-98/Ck/CHN/06	1733/Ck/USA/97	0.00010782
S1133/Ck/CHN/14	1733/Ck/USA/97	0.00275062
T-98/Ck/CHN/06	S1133/Ck/CHN/14	0.00278656
K1600657/Ck/USA/16	V-ARV-SD26/Ck/CHN/20	0.00285444
LY383/Ck/CHN/16	K1600657/Ck/USA/16	0.00285444
LY383/Ck/CHN/16	SDYT2020/Ck/CHN/20	0.00557383
SDYT2020/Ck/CHN/20	V-ARV-SD26/Ck/CHN/20	0.00557383
SDYT2020/Ck/CHN/20	K1600657/Ck/USA/16	0.00828450
LY383/Ck/CHN/16	PHC-2020-0545/Ck/CHN/20	0.00828925
PHC-2020-0545/Ck/CHN/20	V-ARV-SD26/Ck/CHN/20	0.00828925
LY383/Ck/CHN/16	AHZJ19/Ck/CHN/19	0.00832138
AHZJ19/Ck/CHN/19	V-ARV-SD26/Ck/CHN/20	0.00832138
T-98/Ck/CHN/06	MS01/Ck/CHN/19	0.01089808
1733/Ck/USA/97	MS01/Ck/CHN/19	0.01093402
AHZJ19/Ck/CHN/19	K1600657/Ck/USA/16	0.01096017
MS01/Ck/CHN/19	C78/Ck/CHN/13	0.01097022
PHC-2020-0545/Ck/CHN/20	K1600657/Ck/USA/16	0.01099992
S1133/Ck/CHN/14	MS01/Ck/CHN/19	0.01361276
PHC-2020-0545/Ck/CHN/20	SDYT2020/Ck/CHN/20	0.01364743
AHZJ19/Ck/CHN/19	SDYT2020/Ck/CHN/20	0.01375145
AHZJ19/Ck/CHN/19	PHC-2020-0545/Ck/CHN/20	0.01646686
T-98/Ck/CHN/06	C78/Ck/CHN/13	0.01650219
1733/Ck/USA/97	C78/Ck/CHN/13	0.01653813
S1133/Ck/CHN/14	C78/Ck/CHN/13	0.01921687
T-98/Ck/CHN/06	3211-V/Ck/HUN/02	0.02238411
1733/Ck/USA/97	3211-V/Ck/HUN/02	0.02242005
S1133/Ck/CHN/14	3211-V/Ck/HUN/02	0.02509880
01224A/Ck/USA/14	526/Ck/CHN/13	0.02755056
MS01/Ck/CHN/19	3211-V/Ck/HUN/02	0.02777997
C78/Ck/CHN/13	3211-V/Ck/HUN/02	0.03338408
T-98/Ck/CHN/06	01224A/Ck/USA/14	0.04123902
1733/Ck/USA/97	01224A/Ck/USA/14	0.04127496
3211-V/Ck/HUN/02	01224A/Ck/USA/14	0.04150623
S1133/Ck/CHN/14	01224A/Ck/USA/14	0.04395370
MS01/Ck/CHN/19	01224A/Ck/USA/14	0.04663487
T-98/Ck/CHN/06	526/Ck/CHN/13	0.04671569
1733/Ck/USA/97	526/Ck/CHN/13	0.04675163
3211-V/Ck/HUN/02	526/Ck/CHN/13	0.04698290
T-98/Ck/CHN/06	K1600657/Ck/USA/16	0.04782568

1733/Ck/USA/97	K1600657/Ck/USA/16	0.04786162
3211-V/Ck/HUN/02	K1600657/Ck/USA/16	0.04809289
S1133/Ck/CHN/14	526/Ck/CHN/13	0.04943037
S1133/Ck/CHN/14	K1600657/Ck/USA/16	0.05054036
T-98/Ck/CHN/06	LY383/Ck/CHN/16	0.05060824
T-98/Ck/CHN/06	V-ARV-SD26/Ck/CHN/20	0.05060824
1733/Ck/USA/97	LY383/Ck/CHN/16	0.05064418
1733/Ck/USA/97	V-ARV-SD26/Ck/CHN/20	0.05064418
3211-V/Ck/HUN/02	LY383/Ck/CHN/16	0.05087545
3211-V/Ck/HUN/02	V-ARV-SD26/Ck/CHN/20	0.05087545
MS01/Ck/CHN/19	526/Ck/CHN/13	0.05211154
C78/Ck/CHN/13	01224A/Ck/USA/14	0.05223899
MS01/Ck/CHN/19	K1600657/Ck/USA/16	0.05322154
S1133/Ck/CHN/14	LY383/Ck/CHN/16	0.05332292
S1133/Ck/CHN/14	V-ARV-SD26/Ck/CHN/20	0.05332292
**R18-38167/Ck/USA/18**	**K1600657/Ck/USA/16**	**0.05404628**
MS01/Ck/CHN/19	LY383/Ck/CHN/16	0.05600409
MS01/Ck/CHN/19	V-ARV-SD26/Ck/CHN/20	0.05600409
T-98/Ck/CHN/06	SDYT2020/Ck/CHN/20	0.05603830
1733/Ck/USA/97	SDYT2020/Ck/CHN/20	0.05607424
3211-V/Ck/HUN/02	SDYT2020/Ck/CHN/20	0.05630551
R18-38167/Ck/USA/18	LY383/Ck/CHN/16	0.05682884
R18-38167/Ck/USA/18	V-ARV-SD26/Ck/CHN/20	0.05682884
C78/Ck/CHN/13	526/Ck/CHN/13	0.05771566
T-98/Ck/CHN/06	AHZJ19/Ck/CHN/19	0.05871397
1733/Ck/USA/97	AHZJ19/Ck/CHN/19	0.05874991
S1133/Ck/CHN/14	SDYT2020/Ck/CHN/20	0.05875298
T-98/Ck/CHN/06	PHC-2020-0545/Ck/CHN/20	0.05875372
1733/Ck/USA/97	PHC-2020-0545/Ck/CHN/20	0.05878966
C78/Ck/CHN/13	K1600657/Ck/USA/16	0.05882565
3211-V/Ck/HUN/02	AHZJ19/Ck/CHN/19	0.05898118
3211-V/Ck/HUN/02	PHC-2020-0545/Ck/CHN/20	0.05902093
01224A/Ck/USA/14	K1600657/Ck/USA/16	0.06009969
**R18-37308/Ck/USA/18**	**T-98/Ck/CHN/06**	**0.06027485**
R18-37308/Ck/USA/18	1733/Ck/USA/97	0.06031079
R18-37308/Ck/USA/18	3211-V/Ck/HUN/02	0.06054206
S1133/Ck/CHN/14	AHZJ19/Ck/CHN/19	0.06142866
MS01/Ck/CHN/19	SDYT2020/Ck/CHN/20	0.06143416
S1133/Ck/CHN/14	PHC-2020-0545/Ck/CHN/20	0.06146840

The strains displaying the closest evolutionary distance to our isolates, are shown in bold red. The evolutionary distance is shown up to d = 0.06146840.

Table S18. Evolutionary divergence estimates between σNS sequences of ARV isolates.

Species 1	Species 2	Dist
1733/Ck/USA/97	S1133/Ck/CHN/14	0.00014649
T-98/Ck/CHN/06	1733/Ck/USA/97	0.00278027
C78/Ck/CHN/13	S1133/Ck/CHN/14	0.00281473
1733/Ck/USA/97	MS01/Ck/CHN/19	0.00282241
MS01/Ck/CHN/19	S1133/Ck/CHN/14	0.00282241
T-98/Ck/CHN/06	S1133/Ck/CHN/14	0.00285351
C78/Ck/CHN/13	1733/Ck/USA/97	0.00288797
T-98/Ck/CHN/06	MS01/Ck/CHN/19	0.00552944
C78/Ck/CHN/13	MS01/Ck/CHN/19	0.00556390
T-98/Ck/CHN/06	C78/Ck/CHN/13	0.00559500
LY383/Ck/CHN/16	V-ARV-SD26/Ck/CHN/20	0.00827214
LY383/Ck/CHN/16	SDYT2020/Ck/CHN/20	0.00846286
**R18-37308/Ck/USA/18**	**LY383/Ck/CHN/16**	**0.01115762**
R18-37308/Ck/USA/18	SDYT2020/Ck/CHN/20	0.01375624
**R18-37308/Ck/USA/18**	**R18-38167/Ck/USA/18**	**0.01377159**
R18-38167/Ck/USA/18	LY383/Ck/CHN/16	0.01400592
01224A/Ck/USA/14	LY383/Ck/CHN/16	0.01401051
526/Ck/CHN/13	LY383/Ck/CHN/16	0.01493020
01224A/Ck/USA/14	SDYT2020/Ck/CHN/20	0.01653588
R18-38167/Ck/USA/18	SDYT2020/Ck/CHN/20	0.01660454
V-ARV-SD26/Ck/CHN/20	SDYT2020/Ck/CHN/20	0.01666176
R18-37308/Ck/USA/18	01224A/Ck/USA/14	0.01930388
R18-37308/Ck/USA/18	V-ARV-SD26/Ck/CHN/20	0.01935651
526/Ck/CHN/13	SDYT2020/Ck/CHN/20	0.02074305
R18-38167/Ck/USA/18	01224A/Ck/USA/14	0.02215219
R18-38167/Ck/USA/18	V-ARV-SD26/Ck/CHN/20	0.02220482
01224A/Ck/USA/14	V-ARV-SD26/Ck/CHN/20	0.02220940
526/Ck/CHN/13	V-ARV-SD26/Ck/CHN/20	0.02312910
R18-37308/Ck/USA/18	526/Ck/CHN/13	0.02343780
R18-38167/Ck/USA/18	526/Ck/CHN/13	0.02628611
526/Ck/CHN/13	01224A/Ck/USA/14	0.02629069
3211-V/Ck/HUN/02	PHC-2020-0545/Ck/CHN/20	0.03423009
SDYT2020/Ck/CHN/20	K1600657/Ck/USA/16	0.04214728
LY383/Ck/CHN/16	K1600657/Ck/USA/16	0.04514999
PHC-2020-0545/Ck/CHN/20	ARV/AHZJ19/CHN/19	0.04682323
R18-37308/Ck/USA/18	K1600657/Ck/USA/16	0.05044336
22342/Tk/USA/13	D1007/PRT/HUN/08	0.05248436
01224A/Ck/USA/14	K1600657/Ck/USA/16	0.05322300
3211-V/Ck/HUN/02	ARV/AHZJ19/CHN/19	0.05323007

The strains displaying the closest evolutionary distance to our isolates, are shown in bold red. The evolutionary distance is shown up to d = 0.05323007.

Table S19. Accession numbers of sequences used for phylogenetic analysis of ARV coding sequences for genotypic clustering.

Accession No.										Isolate	Year	Country	Abbreviated name*
L1	L2	L3	M1	M2	M3	S1	S2	S3	S4				
OK077993	OK077994	OK077995	OK077996	OK077997	OK077998	OK077999	OK078002	OK078003	OK078004	AHZJ19	2019	China	AHZJ19/Ck/CHN/19
MF183221	MF183212	MF183213	MF183214	MF183215	MF183216	MF183217	MF183218	MF183219	MF183220	LY383	2016	China	LY383/Ck/CHN/16
KY860642	KY860641	KY860640	KY860639	KY860638	KY860637	KY860636	KY860635	KY860634	KY860633	MS01	2017	China	MS01/Ck/CHN/19
MK583331	MK583332	MK583333	MK583334	MK583335	MK583336	MK583337	MK583338	MK583339	MK583340	K1600657	2016	USA	K1600657/Ck/USA/16
KF741696	KF741697	KF741698	KF741699	KF741700	KF741701	KF741702	KF741703	KF741704	KF741705	526	2013	China	526/Ck/CHN/13
KF741706	KF741707	KF741708	KF741709	KF741710	KF741711	KF741712	KF741713	KF741714	KF741715	1733	1997	USA	1733/Ck/USA/97
KX398272	KX398273	KX398274	KX398275	KX398276	KX398277	KX398278	KX398279	KX398280	KX398281	3211-V-02	2002	Hungary	3211-V/Ck/HUN/02
KF741716	KF741717	KF741718	KF741719	KF741720	KF741721	KF741722	KF741723	KF741724	KF741725	C78	2013	China	C78/Ck/CHN/13
MW174784	MW174785	MW174786	MW174787	MW174788	MW174789	MW174790	MW174791	MW174792	MW174793	PHC-2020-0545	2020	China	PHC-2020-0545/Ck/CHN/20
KT428298	KT428299	KT428300	KT428301	KT428302	KT428303	KT428304	KT428305	KT428306	KT428307	Reo/PA/Layer/01224A/14	2014	USA	01224A/Ck/USA/14
KF741756	KF741757	KF741758	KF741759	KF741760	KF741761	KF741762	KF741763	KF741764	KF741765	S1133	2014	China	S1133/Ck/CHN/14
MW394456	MW394457	MW394458	MW394459	MW394460	MW394461	MW394462	MW394463	MW394464	MW394465	SDYT2020	2020	China	SDYT2020/Ck/CHN/20
EU616739	JN641889	EU616738	EU616736	EU616742	EU616743	EF057398	JN641887	EF030499	JN641884	T-98	2006	China	T-98/Ck/CHN/06
MW244842	MW244843	MW244844	MW244845	MW244846	MW244847	MW244848	MW244849	MW244850	MW244851	V-ARV-SD26	2020	China	V-ARV-SD26/Ck/CHN/20
KP173683	KP173684	KP173685	KP173686	KP173687	KP173688	KP173689	KP173690	KP173691	KP173692	Reo/PA/Turkey/22342/13	2013	USA	22342/Tk/USA/13

KR476798	KR476800	KR476799	KR476801	KR476802	KR476803	KR476804	KR476805	KR476806	KR476807	D1007	2008	Hungary	D1007/PRT/HUN/08
JX478250	JX478251	JX478252	JX478253	JX478254	JX478255	JX478256	JX478257	JX478258	JX478259	091	2009	China	091/Dk/CHN/09
AB914760	AB914761	AB914762	AB914763	AB914764	AB914765	AB914766	AB914767	AB914768	AB914769	Pycno-1	2014	Japan	Pycno-1/Bul/JAP/14

*Abbreviated name followed the order of Isolate_name/Host/ Country_of_origin/Collection_year.

Table S20. Accession numbers of sequences used for whole genome alignment.

Accession No.										Isolate	Year	Genogroup (Acc. To Sigma C)	Country	Abbreviated name
L1	L2	L3	M1	M2	M3	S1	S2	S3	S4					
KF741756	KF741757	KF741758	KF741759	KF741760	KF741761	KF741762	KF741763	KF741764	KF741765	S1133	2014	I	China	ARV/S1133/CHN/14
KF741706	KF741707	KF741708	KF741709	KF741710	KF741711	KF741712	KF741713	KF741714	KF741715	1733	1997	I	USA	ARV/1733/USA/97
KR476798	KR476799	KR476800	KR476801	KR476802	KR476803	KR476804	KR476805	KR476806	KR476807	D1007	2008	II	Hungary	D1007/PRT/HUN/08
MW174784	MW174785	MW174786	MW174787	MW174788	MW174789	MW174790	MW174791	MW174792	MW174793	PHC-2020-0545	2020	II	China	ARV/PHC-2020-0545/CHN/20
KP173683	KP173684	KP173685	KP173686	KP173687	KP173688	KP173689	KP173690	KP173691	KP173692	Reo/PA/Turkey/22342/13	2013	II	USA	TARV/22342/USA/13
KT428298	KT428299	KT428300	KT428301	KT428302	KT428303	KT428304	KT428305	KT428306	KT428307	Reo/PA/Layer/01224A/14	2014	III	USA	ARV/01224A/USA/14
KX398272	KX398273	KX398274	KX398275	KX398276	KX398277	KX398278	KX398279	KX398280	KX398281	3211-V-02	2002	IV	Hungary	ARV/3211-V-02/HUN/02
NC_015126	NC_015127	NC_015128	NC_015129	NC_015130	NC_015131	NC_015132	NC_015133	NC_015134	NC_015135	AVS-B	2006	V	USA	AVS-B/Ck/USA/06
MF183221	MF183212	MF183213	MF183214	MF183215	MF183216	MF183217	MF183218	MF183219	MF183220	LY383	2016	VI	China	ARV/LY383/CHN/16
AB914760	AB914761	AB914762	AB914763	AB914764	AB914765	AB914766	AB914767	AB914768	AB914769	Pycno-1	2014	Outgroup	Japan	Pycno-1/Bul/JAP/14

*Abbreviated name followed the order of Isolate_name/Host/ Country_of_origin/Collection_year
